# The genetic landscape of sporadic adult-onset degenerative ataxia: a multi-modal genetic study of 377 consecutive patients from the longitudinal multi-centre SPORTAX cohort

**DOI:** 10.1016/j.ebiom.2025.105715

**Published:** 2025-04-23

**Authors:** Danique Beijer, David Mengel, Demet Önder, Carlo Wilke, Andreas Traschütz, Jennifer Faber, Dagmar Timmann, Sylvia Boesch, Stefan Vielhaber, Thomas Klopstock, Bart P. van de Warrenburg, Gabriella Silvestri, Christoph Kamm, Iselin Marie Wedding, Zofia Fleszar, Florian Harmuth, Claudia Dufke, Bernard Brais, Olaf Rieß, Ludger Schöls, Tobias Haack, Stephan Züchner, David Pellerin, SPORTAX consortium, Friedrich Erdlenbruch, Andreas Thieme, Judith van Gaalen, Christos Ganos, Jun-Suk Kang, Marcus Grobe-Einsler, Ilaria Giordano, Thomas Klockgether, Matthis Synofzik

**Affiliations:** a Division of Translational Genomics of Neurodegenerative Diseases, Hertie-Institute for Clinical Brain Research and Center of Neurology, University of Tübingen, Germany; b German Center of Neurodegenerative Diseases (DZNE), Tübingen, Germany; c German Center for Neurodegenerative Diseases (DZNE), Bonn, Germany; d Center for Neurology, Department of Parkinson's Disease, Sleep and Movement Disorders, University Hospital Bonn, Bonn, Germany; e Department of Neuroradiology, University Hospital Bonn, Bonn, Germany; f Department of Neurology and Center for Translational Neuro- and Behavioral Sciences (C-TNBS), Essen University Hospital, Duisburg-Essen, 45147, Essen, Germany; g Center for Rare Movement Disorders Innsbruck, Department of Neurology, Medical University of Innsbruck, Innsbruck, Austria; h Neurologische Universitätsklinik, Universitätsklinikum Magdeburg A.ö.R., Magdeburg, Germany; i Department of Neurology with Friedrich-Baur-Institute, LMU University Hospital of Ludwig-Maximilians-Universität München, 80336, Munich, Germany; j German Center for Neurodegenerative Diseases (DZNE), Munich, Germany; k Munich Cluster for Systems Neurology (SyNergy), Munich, Germany; l Department of Neurology, Radboud University Medical Center, 6525, Nijmegen, the Netherlands; m Department of Neurosciences, Università Cattolica del Sacro Cuore, Rome, Italy; n UOC Neurologia Dipartimento Neuroscienze, Fondazione Policlinico Universitario A Gemelli IRCCS, Organi Di Senso e Torace, Rome, Italy; o Department of Neurology, University of Rostock, Rostock, Germany; p Department of Neurology, Oslo University Hospital, Oslo, Norway; q Department of Neurodegenerative Diseases and Hertie-Institute for Clinical Brain Research and Center of Neurology, University of Tübingen, Germany; r Institute of Medical Genetics and Applied Genomics, University of Tübingen, Tübingen, Germany; s Department of Neurology and Neurosurgery, Montreal Neurological Hospital and Institute, McGill University, Montreal, QC, Canada; t Department of Human Genetics, McGill University, Montreal, QC, Canada; u Department of Human Genetics and John P. Hussman Institute for Human Genomics, University of Miami Miller School of Medicine, Miami, 33136, FL, USA; v Department of Neurology, University Hospital Bonn, Bonn, Germany

**Keywords:** Sporadic ataxia, Adult-onset ataxia, Multiple system atrophy, Genomics, SCA27B, CANVAS, Disease trajectories, Genetic testing, Prospective cohort

## Abstract

**Background:**

While most sporadic adult-onset neurodegenerative diseases have only a minor monogenic component, given several recently identified late adult-onset ataxia genes, the genetic burden may be substantial in sporadic adult-onset ataxias. We report systematic mapping of the genetic landscape of sporadic adult-onset ataxia in a well-characterised, multi-centre cohort, combining several multi-modal genetic screening techniques, plus longitudinal natural history data.

**Methods:**

Systematic clinico-genetic analysis of a prospective longitudinal multi-centre cohort of 377 consecutive patients with sporadic adult-onset ataxia (SPORTAX cohort), including clinically defined sporadic adult-onset ataxia of unknown aetiology (SAOA) (n = 229) and ‘clinically probable multiple system atrophy of cerebellar type’ (MSA-C_cp_) (n = 148). Combined GAA-*FGF14* (SCA27B) and *RFC1* repeat expansion screening with next-generation sequencing (NGS) was complemented by natural history and plasma neurofilament light chain analysis in key subgroups.

**Findings:**

85 out of 377 (22.5%) patients with sporadic adult-onset ataxia carried a pathogenic or likely pathogenic variant, thereof 67/229 (29.3%) patients with SAOA and 18/148 (12.2%) patients meeting the MSA-C_cp_ criteria. This included: 45/377 (11.9%) patients with GAA-*FGF14*_≥250_ repeat expansions (nine with MSA-C_cp_), 17/377 (4.5%) patients with *RFC1* repeat expansions (three with MSA-C_cp_), and 24/377 (6.4%) patients with single nucleotide variants (SNVs) identified by NGS (six with MSA-C_cp_). Five patients (1.3%) were found to have two relevant genetic variants simultaneously (dual diagnosis).

**Interpretation:**

In this cohort of sporadic adult-onset ataxia, a cohort less likely to have a monogenic cause, a substantial burden of monogenic variants was identified, particularly GAA-*FGF14 and RFC1* repeat expansions. This included a substantial share of patients meeting the MSA-C_cp_ criteria, suggesting a reduced specificity of this clinical diagnosis and potential co-occurrence of MSA-C plus a second, independent genetic condition. These findings have important implications for the genetic work-up and counselling of patients with sporadic ataxia, even when presenting with MSA-like features. With targeted treatments for genetic ataxias now on the horizon, these findings highlight their potential utility for these patients.

**Funding:**

This work was supported by the Clinician Scientist programme “PRECISE.net” funded by the Else Kröner-Fresenius-Stiftung (to DM, AT, CW, OR, and MS), by the 10.13039/501100001659Deutsche Forschungsgemeinschaft (as part of the PROSPAX project), and by the Canadian Institutes of Health Research and the 10.13039/501100024730Fondation Groupe Monaco. Support was also provided by Humboldt Research Fellowship for Postdocs and the Hertie-Network of Excellence in Clinical Neuroscience and a Fellowship award from the 10.13039/501100000024Canadian Institutes of Health Research.

**Research in context**

**Evidence before this study**

Most sporadic adult-onset neurodegenerative diseases have a minor monogenic component, which complicates mechanistic understanding and the development of targeted mechanistic therapies for these conditions. However, recent discoveries of several adult-onset ataxia genes — some with onset even in late adulthood — suggest a potential for a substantial monogenic contribution in sporadic adult-onset ataxias. Earlier genetic screening studies in sporadic ataxias indicated a significant contribution of monogenic causes, but were limited by their lack of leveraging of any next-generation sequencing (NGS) approach; investigated only *one* gene (e.g., *RFC1*; or GAA-*FGF14*); did not maximise for exclusion of common autosomal-dominant causes by systematically excluding positive family history in parental generations and prior exclusion of common CAG-repeat spinocerebellar ataxias; and/or did not investigate a multi-centre cohort.

**Added value of this study**

This study provides in-depth insights into the genetic architecture of sporadic adult-onset ataxia by systematically mapping its genetic landscape through a multimodal genetic approach that combines NGS with *RFC1* and GAA-*FGF14* genotyping in a large, well-defined, strictly consecutive cohort, as well as longitudinal natural history data and plasma neurofilament light chain (NfL) analyses in relevant key subgroups. Our findings reveal a substantial burden of monogenic variants in patients with sporadic adult-onset ataxia (22%), particularly highlighting the prevalence of GAA-*FGF14* and *RFC1* repeat expansions. Moreover, they demonstrate that a noteworthy share of patients (12%) meeting the criteria of ‘clinically probable multiple system atrophy cerebellar type’ (MSA-C_cp_), according to the 2nd consensus multiple system atrophy (MSA) diagnostic criteria, have a monogenic basis. This suggests a reduced specificity of this clinical diagnostic criteria for MSA mimics and the potential co-occurrence of MSA plus a second, independent genetic condition (dual diagnosis).

**Implications of all the available evidence**

The significant monogenic burden identified in this study demonstrates the need for comprehensive genetic investigations in patients with apparently “sporadic” adult-onset ataxia. This also applies to patients meeting the 2nd consensus diagnostic criteria of ‘clinically probable MSA’. The clinical presentation as “sporadic” ataxia — even for late onset forms — can be explained, *inter alia*, by late adult-onset ataxias with dynamic repeat expansions (e.g., *GAA-FGF14*) or with autosomal-recessive inheritance (e.g., *RFC1*). Genetic screening protocols in sporadic adult-onset ataxia should routinely include tests, in particular for GAA-*FGF14* and *RFC1* repeat expansions, to improve diagnostic accuracy, genetic counselling, and access to targeted treatments — a key opportunity which would otherwise be critically missed in these apparently “sporadic” patients.

## Introduction

In contrast to most other sporadic adult-onset neurodegenerative diseases (e.g., Alzheimer's disease, amyotrophic lateral sclerosis (ALS), or Parkinson's disease) in which aetiology is more complex (includes environmental factors), sporadic adult-onset ataxias seem to have a higher monogenic burden in.^1^, ^2^, ^3^ In particular the recent identification of genes prominently associated with late adult-onset sporadic ataxias—such as GAA*-FGF14*- (SCA27B)^4^, ^5^, ^6^ or *RFC1*-related ataxia^7^, ^8^, ^9^ – indicates that the contribution of monogenic causes underlying sporadic adult-onset ataxia could be substantial. This might also include patients with sporadic adult-onset ataxia meeting the 2nd consensus MSA diagnostic criteria of ‘clinically probable MSA-C’ (MSA-C_cp_),^10^ as instances of single patients with an MSA-C-like phenotype have been reported for several adult-onset ataxia genes (e.g., *RFC1*,^8^^,^^11^^,^^12^ GAA*-FGF14*^13^ or *SPG7*^14^).

Systematically unravelling the genetic landscape underlying sporadic adult-onset ataxia, which includes both patients with sporadic adult-onset ataxia of unknown aetiology (SAOA) and patients with multiple system atrophy of cerebellar type (MSA-C), has important implications beyond genetic work-up and counselling of these patients. With targeted treatments for genetic ataxias now on the horizon,^15^, ^16^, ^17^ and for *GAA-FGF14*--associated ataxia already in sight by an FDA-approved drug (4-Aminopyridine^6^^,^^18^^,^^19^), it is increasingly relevant to accurately diagnose these patients with the underlying genetic cause, thereby providing access to future targeted treatments.

Here, we performed a systematic multimodal clinico-genetic analysis of the genetic landscape underlying sporadic adult-onset ataxia, leveraging a large prospective longitudinal multi-centre cohort of 377 consecutive patients with sporadic adult-onset ataxia (SPORTAX cohort).^20^^,^^21^ In this analysis, we combined complementary genetic screening techniques (GAA-*FGF14* and *RFC1* repeat expansion screening, plus next-generation sequencing), complemented by longitudinal natural history data and plasma neurofilament light chain (NfL) analysis in subgroups of interest. We hypothesised that a substantial share of patients with sporadic adult-onset ataxia– despite by their sporadic, adult-onset presentation being less likely to have a genetic cause—might carry a pathogenic or likely pathogenic variant, in particular GAA*-FGF14* or *RFC1* expansions. This might also include a substantial share of patients meeting the 2nd consensus MSA criteria for MSA-C_cp_,^10^ as these criteria might have a reduced specificity against the background of genetic MSA-mimics and pathogenic variants might also simply co-occur as a second, independent condition to MSA.

## Methods

### Study design, cohort characteristics, and patients

SPORTAX is a registry-inventoried prospective longitudinal European multi-centre observational cohort study (fourteen sites, from five different European countries; Charité University Hospital, Berlin (Germany); University Hospital Bonn, Bonn (Germany); Essen University Hospital, Essen (Germany); University of Frankfurt, Frankfurt am Main (Germany); University Medical Center Hamburg, Hamburg (Germany); Medical University of Innsbruck, Innsbruck (Austria); Universitätsklinikum Magdeburg A.ö.R., Magdeburg (Germany); University Hospital of Ludwig-Maximilians-Universität München, München (Germany); Federico II University, Naples (Italy); Radboud University Medical Center, Nijmegen (the Netherlands); Oslo University Hospital, Oslo (Norway); Universitario “A. Gemelli” IRCCS, Rome (Italy); University of Rostock, Rostock (Germany); Hospital Tübingen, Tübingen (Germany)). SPORTAX investigated the characteristics and evolution of sporadic adult-onset ataxia, including genetics, clinical outcome measures, and fluid biomarkers, with recruitment having started on April 1, 2010.^20^^,^^21^ Inclusion criteria of the predefined study protocol had been designed to specifically target sporadic (late-)adult-onset degenerative ataxia: (i) progressive ataxia with age of ataxia onset >40 years; (ii) informative and negative family history (no similar disorders in first- and second-degree relatives; parents older than 50 years, or, if not alive, age at death of more than 50 years, no consanguinity of parents); (iii) negative molecular genetic testing for Friedreich's ataxia (FRDA), spinocerebellar ataxia type 1 (SCA1), SCA2, SCA3, SCA6, and Fragile X Messenger Ribonucleoprotein 1 (FMR1) pre-mutation; and (iv) no established acquired cause of ataxia. No further ethnic inclusion/exclusion criteria were applied, thus aiming to make our cohort representative of the broader European population as seen in ataxia referral centres across Europe.

Longitudinal follow-up assessments were carried out at each visit of a patient, if possible, on an annual basis. Phenotyping and biomarker assessments including prospective longitudinal follow-up assessments were performed blinded to the genotype results of the current study.

Patients were classified with clinical MSA-C if they fulfilled the diagnostic criteria for MSA-C_cp_ according to the 2nd consensus MSA diagnostic criteria at least at the last visit.^10^ These diagnostic MSA criteria were used as they were the only criteria available at time of study enrolment and data capture. Since subjects were enrolled in the study and longitudinal data captured from 2010 until 2020, they could not have been assessed with the more recent Movement Disorders Society MSA criteria published in 2022.^22^ The remaining patients were classified with clinical SAOA. Further details on criteria and workup of the SPORTAX cohort can be found in Oender et al., 2022.^20^

DNA extraction was performed on peripheral blood samples obtained from patients. DNA availability did not significantly correlate with any other study metrics, except study centre. Overall, DNA availability per centre is a systematic difference that has, however, not incurred any bias in our cohort or genetic findings, thus our study outcomes (see Statistics).

For 377/436 patients of the SPORTAX cohort, sufficient quantity and quality of DNA was available, which included 229 patients with SAOA (60.7%) and 148 patients with MSA-C_cp_ (39.3%). Genetic testing of these patients included complementary genetic screening techniques: GAA-*FGF14* (SCA27B) repeat expansion screening, *RFC1* repeat expansion screening (initially associated primarily with cerebellar ataxia, neuropathy and vestibular areflexia syndrome (CANVAS)), next-generation sequencing (Supplement 1). Patients were investigated by each of these techniques when DNA was available (356 individuals by GAA*-FGF14* repeat expansion screening, 360 individuals by *RFC1* repeat expansion screening, 301 individuals by next-generation sequencing (NGS)), even if one of these techniques already rendered a positive result, thus allowing to test also for possible dual genetic diagnoses and to include/exclude also other (potentially more convincing) genetic variants.

Preliminary earlier results of a genetic analysis of the SPORTAX cohort has previously been published,^20^ however this previous analysis (i) used genetic tests to *exclude* patients from the respective subsequent analyses; (ii) was only preliminary in nature; and (iii) lacked several genetic analyses employed in the current multi-modal genetic study (for example, preliminary exome sequencing analysis without GAA-*FGF14* genotyping).

### Ethics

The SPORTAX study was approved by the ethics committee of University Tübingen (AZ 598/2011BO1). All participants provided written informed consent. This study is registered with ClinicalTrials.gov (NCT02701036).

### GAA-*FGF14* expansion screening

In brief, the intronic *FGF14* repeat locus was amplified by long-range PCR and the number of repeat units was subsequently determined by capillary electrophoresis of fluorescent long-range PCR amplification products, using methodology and primers as previously described by Pellerin et al.^4^ Results of fragment length analysis were confirmed by agarose gel electrophoresis of PCR amplification products. Additionally, bidirectional repeat-primed PCRs targeting the 5′-end and the 3′-end of the locus were used to ascertain the presence of a GAA repeat expansion. GAA repeat expansions ≥250 repeat units were considered pathogenic.^4^^,^^6^ GAA repeat expansions 200–250 repeat units were considered of potential pathogenic relevance (given recent observations that GAA 200–250 repeat units might cause GAA-*FGF14* ataxia syndromes).^19^

### *RFC1* expansion screening

Genetic screening for *RFC1* repeat expansions was performed as previously described assessing two motifs, (AAAAG) and (AAGGG), using repeat-primed PCR and an additional flanking PCR.^23^ In brief, the intronic genomic region of the *RFC1* expansion was amplified from genomic DNA using a fluorescence-labelled PCR primer. The number of 5 bp repeat motifs was calculated from the length of the alleles as determined by capillary electrophoresis (ABI3730, Applied Biosystems) allowing for an exact determination of repeat numbers up to 115 repeats. In addition, a repeat-primed PCR to target the frequent pathogenic motive (AAGGG, primer sequences from Cortese et al.,^7^ and the non-pathogenic motive (AAAAG, primer sequences from^5^) was performed. The presence of biallelic AAGGG *RFC1* expansion was confirmed if flanking PCR did not show an amplifiable fragment and the repeat-primed PCR for AAAAG did not show peaks, while the repeat-primed PCR for the AAGGG showed the typical sawtooth peak pattern.

### Next-generation sequencing and analysis

#### Next-generation sequencing

The high-coverage custom HaloPlex gene panel (Agilent, Santa Clara, CA) was run on a NextSeq500 sequencer (Illumina, San Diego, CA) with paired-end 2 × 150 bp sequencing (671 kb target size) as previously described.^21^ The mean vertical coverage was 413 reads, and a minimal coverage of 20 reads was achieved for 98.8% of the target region. Bioinformatic analysis of the variants were described previously.^21^ Exome sequencing (ES) was run on the Illumina NovaSeq 6000 platform using the Agilent SureSelectXT library preparation kit and the SureSelect Human All Exon V7 enrichment kit (Q30-value: 92.34%). Reads were aligned with BWA. Variants were called with Picard and FREEBAYES and annotated with ANNOVAR as part of the GENESIS platform.^24^

#### Targeted gene analyses

201 genes known to be associated with ataxia (Gene set 1, Supplement 2) were screened by NGS, either with a high-coverage large-scale NGS panel (n = 154 subjects) or by exome sequencing (ES) (n = 117 subjects), or both (n = 30 subjects). For exome sequencing, an additional 186 ataxia-overlap disease genes (Gene set 2, Supplement 2), as well as a large gene set of 957 disease genes associated with other types of neurodegenerative disease (Gene set 3, Supplement 2) were evaluated. Data was processed as described previously^20^^,^^24^ (Supplement 3). Called variants were filtered for read quality, read depth, population frequency, and for variant effect and annotated with available information from mutation database ClinVar. Pathogenicity of variants was determined by semi-automated application of ACMG criteria using InterVar with manual correction where needed.^25^, ^26^, ^27^

Subjects were classified as having a genetic diagnosis based on the pathogenic likelihood of the respective variants and the phenotypic match. That is, subjects and their diagnoses were classified as: (i) definitive genetic diagnosis, if having a pathogenic or likely pathogenic variant (based on application of ACMG criteria) and a phenotype typical of the genetic variant; (ii) probable genetic diagnosis, if having a pathogenic or likely pathogenic variant (based on application of ACMG criteria) and a phenotype broadly compatible with the genetic variant; (iii) unclear in cases where a pathogenic or likely pathogenic variant (based on application of ACMG criteria) was found, but the phenotype was not a characteristic match or additional genetic studies would be needed to provide final proof; (iv) no genetic diagnosis (all other subjects).

### Plasma neurofilament light chain analyses

Plasma NfL was determined as described previously.^20^ Study sites collected EDTA plasma samples that were frozen at −80 °C within 1 h post collection and analysed without any further freeze–thaw cycle. Plasma levels of NfL were quantified using the Simoa NF-light Advantage kit (Lot 502,183) on an Quanterix HD1 analyser (Quanterix, Billerica, MA). All assays were performed by the same operator blinded to sample identity. EDTA plasma was centrifuged at 14,000×*g* for 4 min, and the upper 90% transferred to the assay plate. Samples (dilution factor 1:4 in sample buffer) and calibrators were analysed in duplicate. Two internal control samples were assessed both at the start and end of an assay plate. The repeatability was 3.7% (sample 1) and 5.7% (sample 2). The inter-assay variance between the runs across 5 days was 3.1% (sample 1) and 4.8% (sample 2). Cross-sectional evaluation of NfL levels always used the first available NfL level at a subjects first visit in the SPORTAX study.

### Statistics

The statistical analyses were conducted using GraphPad Prism, version 10.1.2 (La Jolla, CA, USA), and Stata, version 17.0 (College Station, TX, USA). Normal distribution of data was assessed by visually inspecting histograms and Quantile–Quantile plots. Fisher's exact tests were performed to assess the statistical significance of the difference in the yield of genetic diagnoses (categorical) between the SAOA and MSA-C_cp_ subgroups, where relevant. Clinical and cohort features (e.g., sex, age at onset, disease severity) were compared between groups using student's t-tests (for continuous variables) or Fisher's exact tests (for categorical variables). A comparison for the presence of GAA-*FGF14* intermediate repeat expansion length (200–249 GAA repeats) was performed using a Fisher's exact test with the control population as described in Pellerin et al. (2024).^28^ Differences in NfL levels between the non-genetic MSA-C_cp_ group compared to the GAA-*FGF14* and *RFC1* MSA-C_cp_ and SAOA groups were examined using the Kruskal–Wallis H test followed by Dunn's post-hoc test. NfL levels are age-dependent, with higher levels observed in older individuals. To account for this, we also modelled longitudinal plasma NfL levels in the non-genetic MSA-C_cp_ group compared to the GAA-*FGF14* and RFC1 MSA-C_cp_ and SAOA groups using a linear mixed effect model (LMEM) that included baseline age as a covariate. The interpretation of the statistical results from this model was similar to that of the original simpler cross-sectional comparison. LMEM with an unstructured covariance matrix was employed to investigate the relationship between the response variable (SARA—Scale for the Assessment and Rating of Ataxia) and predictor variables. The LMEMs were adjusted for age of disease onset and sex, and included an interaction between time since disease onset and diagnostic group as well as random intercepts and slopes nested within subjects. Model building began with the formulation of a null model, and covariates and additional multilevel factors were systematically introduced to assess their impact on the response variable. Model fit was evaluated using the Akaike Information Criterion (AIC) and Bayesian Information Criterion (BIC), with lower values indicating better fit. Additionally, the interclass correlation coefficient (ICC) was computed to assess the proportion of total variance attributable to level-2 variability Additionally, the interclass correlation coefficient (ICC) was computed to assess the proportion of total variance attributable to level-2 variability. Homoscedasticity of level-1 residuals, which represent within-group deviations from the predicted values, was assessed using residual plots, was assessed using residual plots, while symmetry of total residuals was examined through visual inspection of histograms. Individual subjects' longitudinal annualised disease progression was determined by the slope of the line through SARA scores from their first and their last available assessment. We report p-values that are unadjusted for multiple comparisons due to the exploratory nature of our study. The significance threshold was set p < 0.05.

Subgroup analysis for the centres was carried out by analysing differences between the three largest contributing sites, as the large variability of patients included per centre–with low contributions from some centres–did not allow for a meaningful per-centre analysis. No correlations with the study sites (p-values >0.1) for any continuous data patient characteristic (age at onset, age at visit, SARA score, NfL concentration) or categorical value (sex, phenotypic subgroup and genetic diagnosis) was found. DNA availability was an exception, indicating only that specific centres were more likely to sample for DNA, without selecting a distinct patient population.

### Sex and race/ethnicity reporting

Sex data were collected based on self-reporting by participants or as recorded in their medical records. Race and ethnicity data were not collected as part of our study. Patients were included in the study solely based on meeting the inclusion criteria, regardless of sex and race/ethnicity. No specific analyses were designed to assess differences in genetic findings by sex.

### Role of funders

Funders had no role in the study design, data collection, analysis, interpretation or writing of this manuscript.

## Results

The analysis of 377 patients with sporadic adult-onset ataxia (213 male, 164 female) from the SPORTAX cohort included 229 (60.7%) SAOA patients (131 male, 81 female) and 148 (39.3%) patients meeting the 2nd consensus MSA-C_cp_ diagnostic criteria (MSA-C_cp_ patients) (82 male, 66 female). A detailed overview of the cohort characteristics, including demographic and clinical features, is provided in Supplement 4. (Supplement 4; cohort characteristics). Median disease duration at time of the most recent follow-up within the cohort was 6.7 years (IQR 4.8–10.7) (SAOA: 7.7 years (IQR 4.9–13.1), MSA-C_cp_: 6.1 years (IQR 4.6–7.5); p < 0.0001, student's t-test), and significantly shorter in MSA-C_cp_. The median disease severity at the same assessment was 15 SARA points (IQR 11–20) (SAOA: 13 SARA points (IQR 9.5–17), MSA-C_cp_: 19 SARA points (IQR 14–24.5); p < 0.0001, student's t-test) and significantly higher in MSA-C_cp_.

**Fig. 1:**
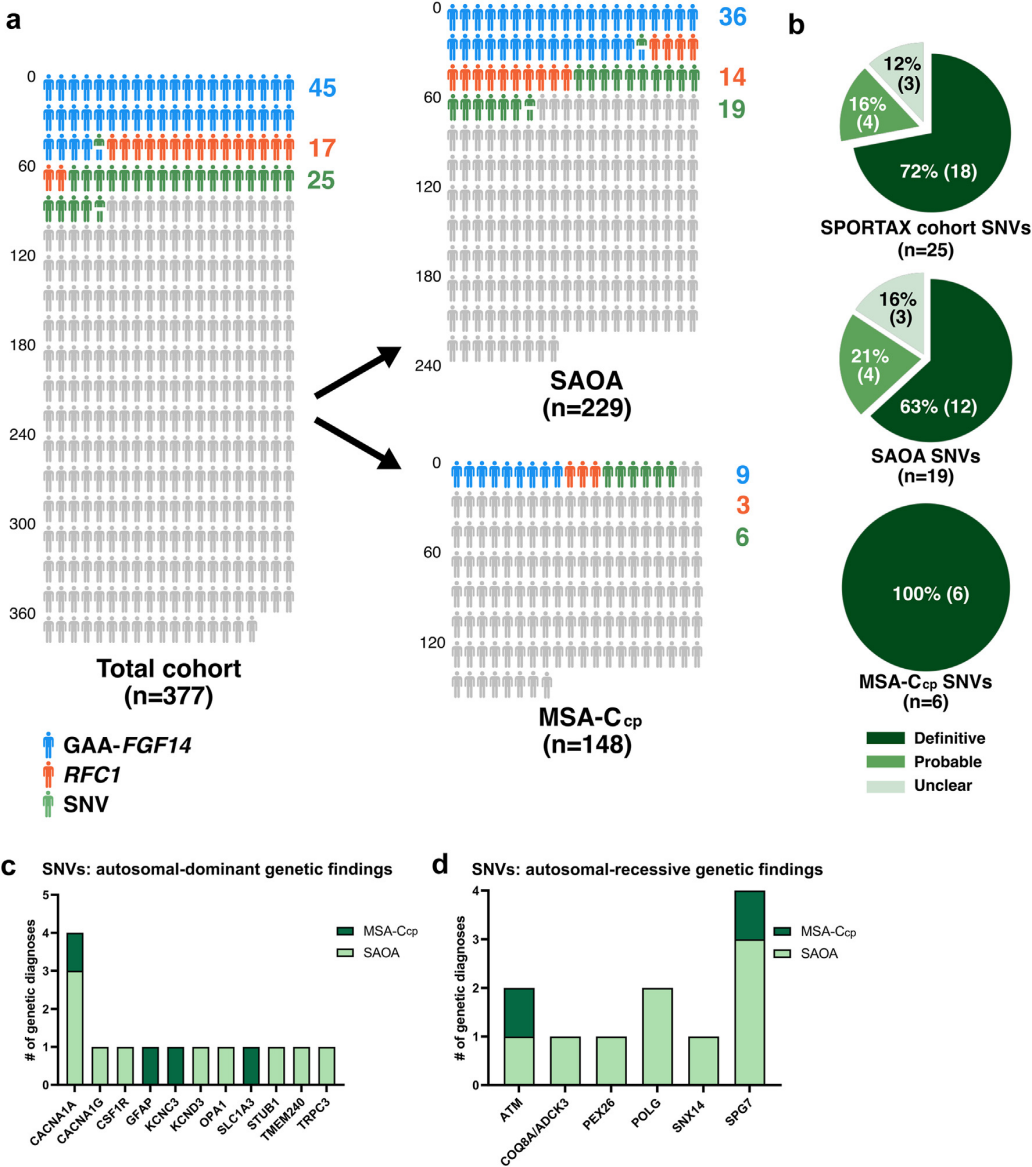
**Genetic landscape of sporadic adult-onset degenerative ataxia**. (a) 377 individuals with sporadic adult-onset ataxia from the SPORTAX cohort underwent genetic testing (229 SAOA patients and 148 MSA-C_cp_ patients). *FGF14* (GAA)_≥250_ repeat alleles were identified in 45 individuals (blue); biallelic *RFC1 (*AAGGG*)* repeat expansions were identified in 17 individuals (orange); 25 different definitive, probably or unclear genetic diagnoses through SNVs were identified in 24 individuals (green); one individual has two relevant SNV findings. Two individuals had multiple relevant genetic findings for the ataxia phenotype demonstrated by split pictographs (P25 and P40). (b) number and percentage of SNV findings divided by phenotypic subtype and SNV interpretation (definitive/probable/unclear). (c, d) Number of individuals with a relevant genetic finding of SNVs (n = 25, definitive, probable and unclear) in respective autosomal-dominant (n = 14) (c) or autosomal-recessive (n = 11) (d) genes. Only genetic SNVs of the definitive, probably and unclear categories are shown here as based on the interpretation of the link between variants and phenotype, separated by gene in MSA-C_cp_ individuals (dark green) and SAOA individuals (light green). Patients with MSA-C_cp_ and meeting the criteria of clinically probable multiple system atrophy cerebellar type; SAOA, sporadic adult-onset ataxia of unknown aetiology.

Overall, a pathogenic or likely pathogenic variant was identified in 85/377 individuals, rendering an overall genetic yield of 22.5% in this cohort (Fig. 1a). Of the identified SNVs 72% were definitive in classification (Fig. 1b), and could further be subdivided into autosomal dominant (Fig. 1c) and autosomal recessive (Fig. 1d) variants. Per clinical subgroup, 67/229 (29.3%) SAOA patients and 18/148 (12.2%) MSA-C_cp_ patients carried a pathogenic or likely pathogenic variant (Fig. 1a).

### GAA-*FGF14* repeat expansions in sporadic ataxia

Heterozygous *FGF14* (GAA)_≥250_ repeat alleles were identified in 45/377 individuals (11.9%), thereof 36/229 patients (15.7%; 257–483 GAA repeat units) in the SAOA cohort and 9/148 (6.1%; 273–409 GAA repeat units) in the MSA-C_cp_ cohort (Supplement 5, pathogenic FGF14-GAA repeat expansion sizes). Of the nine *FGF14* (GAA)_≥250_ patients with MSA-C_cp_ phenotype, the MSA_cp_ criterion of autonomic dysfunction was met in 5/9 patients with orthostatic hypotension (>30 mmHG systolic or 15 mmHG diastolic), 2/9 patients with genitourinary dysfunction according to the MSA_cp_ criteria (urinary incontinence, with erectile dysfunction in males) and 2/9 patients with both autonomic dysfunctions (Table 1, for additional phenotypic information see Supplement 10). Age of onset was 61 years (IQR 56–63), current age 68 years (IQR 63–72), and disease duration 5.2 years (IQR 3.9–7.2) in the GAA-*FGF14* MSA-C_cp_ group. Each of which was not significantly higher than in the GAA-*FGF14* SAOA group (age of onset: 60 years (IQR 57–68.3), current age: 73 (IQR 67.8–79), disease duration: 9.5 years (6.5–15.3); p > 0.05 student's t-test), indicating that it was not a potentially higher age and/or disease duration that might have contributed to the MSA_cp_ phenotype in those patients with GAA-*FGF14* expansions meeting the MSA-C_cp_ criteria. 5/9 GAA-*FGF14* MSA-C_cp_ subjects showed downbeat nystagmus (DBN), a sign characteristic for GAA-*FGF14* ataxia (while much less frequent in MSA-C) (Supplement 10). This indicates that in at least some subjects with GAA-*FGF14* MSA-C_cp_ the MSA-like phenotype may present a phenotypic cluster along the continuous phenotypic spectrum of *GAA-FGF14*-ataxia. In turn, however, 2/9 subjects with GAA-*FGF14* MSA-C_cp_ (including one subject with DBN) also showed signs characteristic of MSA-C like erectile dysfunction and polyminimyoclonus (patient 6) or stridor (patient 7) (Supplement 10), which are very untypical for patients with GAA-*FGF14* ataxia. This indicates that in at least some patients with GAA-*FGF14* MSA-C_cp_, MSA-C might also be present as a coexistent, second independent condition.

**Table 1:** Phenotype of patients with genetic findings meeting the MSA-C_cp_ criteria (for more phenotypic details, see Supplement 8 and Supplement 10 and 11).

Individual#	Sex	Age at onset	Age at assessment	SARA	NfL levels (pg/mL)	Genitourinary dysfunction for MSA-C_cp_ met^a^	Orthostatic hypotension criterion for MSA-C_cp_ met^b^	Downbeat-nystagmus on fixation
**GAA-*FGF14***_***≥250***_**MSA-C_cp_ patients**								

P1	F	62	65	9	NA	no	yes	yes
P2	F	56	61	25	NA	yes	yes	no
P3	M	61	63	19	NA	no	yes	no
P4	M	67	72	8	NA	no	yes	yes
P5	F	63	70	17.5	13.5	yes	yes	yes
P6	M	55	59	19	NA	no	yes	no
P7	M	60	68	15.5	12.3	yes	no	yes
P8	M	77	77	11	14.5	no	yes	no
P9	M	50	76	7.5	14.8	yes	no	yes

Individual #	Sex	Age at onset	Age at assessment	SARA	NfL levels (pg/mL)	Genitourinary dysfunction for MSA-C_cp_ met^a^	Orthostatic hypotension criterion for MSA-C_cp_ met^b^	≥Two out of three additional typical clinical CANVAS signs^c^
***RFC1* MSA-C_cp_ patients**								

P10	F	46	61	14	NA	yes	no	yes
P11	M	47	61	9.5	16	no	yes	yes
P12	F	49	64	22.5	27.7	yes	no	yes

Individual #	Sex	Age at onset	Age at assessment	SARA	NfL levels (pg/mL)	Genitourinary dysfunction for MSA-C_cp_ met^a^	Orthostatic hypotension criterion for MSA-C_cp_ met^b^	–
**SNV MSA-C_cp_ patients**								

P13	M	40	42	6	NA	yes	no	–
P14	F	51	54	10	10.2	yes	no	–
P15	F	65	76	26	22.5	yes	no	–
P16	F	48	54	17.5	NA	yes	no	–
P17	F	54	71	9.5	9.4	yes	no	–
P18	M	57	60	18.5	NA	yes	no	–

Abbreviations: #, number; CANVAS, Cerebellar ataxia, neuropathy and vestibular areflexia syndrome; MSA-C_cp_, patients meeting the criteria of clinically probable multiple system atrophy cerebellar type; SARA, Scale for the assessment and rating of ataxia; SNV, single nucleotide variant. MSA criteria according to the 2nd consensus MSA diagnostic criteria.

a Urinary incontinence (inability to control the release of urine from the bladder, with erectile dysfunction in males).

b By at least 30 mmHg systolic or 15 mmHg diastolic within 3 min of standing.

c Presence of two or more of the following clinical signs: sensory neuropathy/ganglionpathy, impaired head impulse test, chronic cough).

**Fig. 2:**
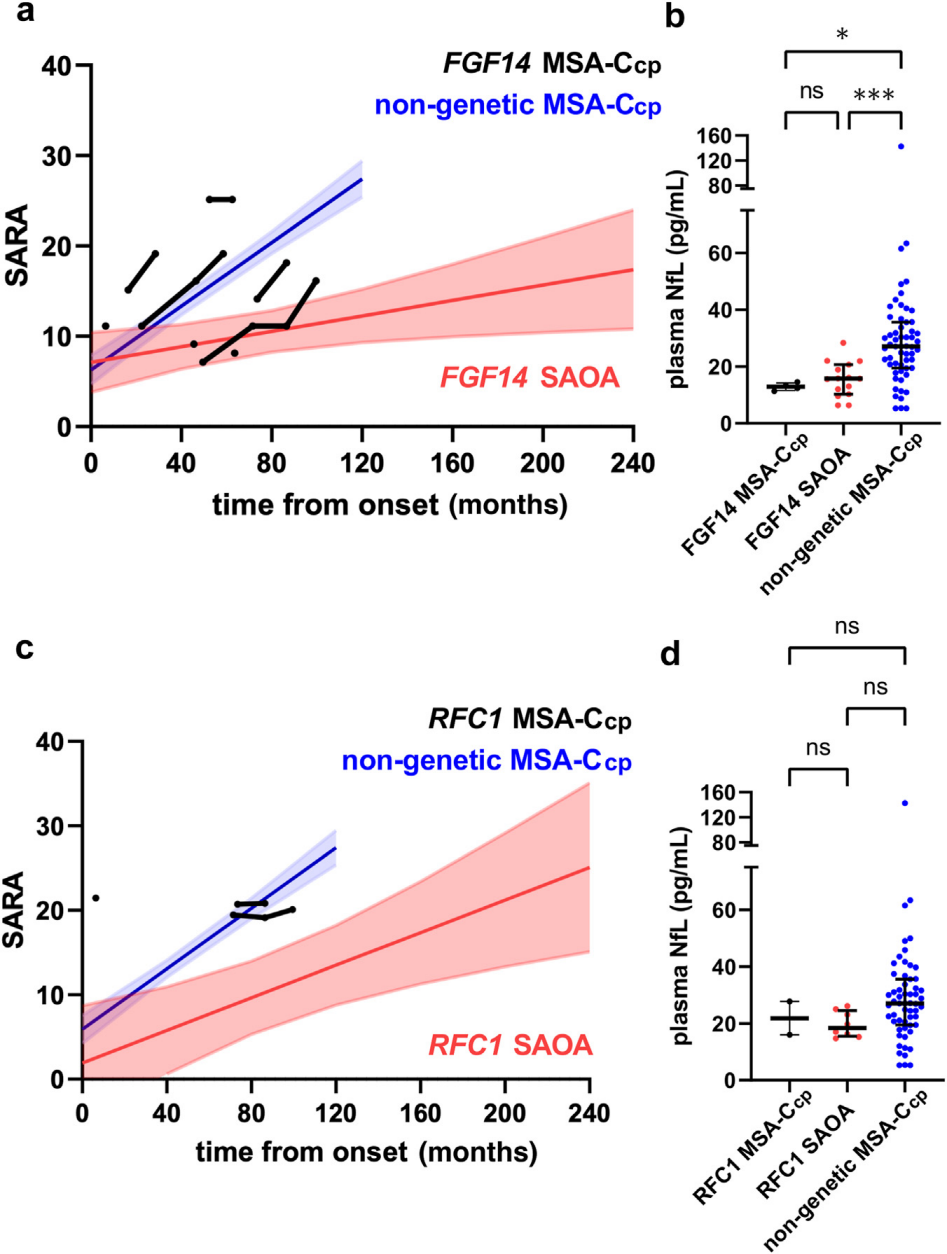
**Progression of disease severity (SARA), and cross-sectional NfL assessments in different ataxia disease groups**. (a, c) Progression of disease severity as calculated by a linear mixed effect model (LMEM) combining cross-sectional and longitudinal SARA scores, plotted against time from onset, estimated trajectories per disease group with 95% CIs, GAA*-FGF14* (a) (*FGF14* MSA-Ccp n = 9, non-genetic MSA-Ccp n = 72, *FGF14* SAOA n = 36), *RFC1* (c) (*RFC1* MSA-Ccp n = 3, non-genetic MSA-Ccp n = 72, *RFC1* SAOA n = 14). (b, d) Cross-sectional plasma NfL levels at first visit per disease group, GAA*-FGF14* (b) (*FGF14* MSA-Ccp n = 4, non-genetic MSA-Ccp n = 58, *FGF14* SAOA n = 14), *RFC1* (d) (*RFC1* MSA-Ccp n = 2, non-genetic MSA-Ccp n = 58, *RFC1* SAOA n = 8). Patients with MSA-C_cp_ patients meeting the criteria of clinically probable multiple system atrophy cerebellar type; SAOA, sporadic adult-onset ataxia of unknown aetiology. Each point represents an individual subject and means ± SEM are indicated. Differences between groups were assessed Kruskal Wallis H test followed by Dunn's post hoc test, ∗p < 0.05, ∗∗∗p < 0.001, ns is non-significant.

The disease progression rate in patients with MSA-C_cp_ and GAA-*FGF14* expansions (n = 9), calculated by the LMEM which allows consideration of both cross-sectional and longitudinal datapoints, was 0.93 ± 0.51 SARA points/year (for all intraindividual progression plots, see Fig. 2a). If assessed on a group level, this disease progression was substantially slower than the disease progression of MSA-C_cp_ patients in whom no genetic variant was found (2.11 ± 0.16 SARA points/year, p = 0.03, n = 72), and more similar to the patients with SAOA and GAA-*FGF14* expansions (0.51 ± 0.22 SARA points/year, p = 0.45, n = 36). Yet, on an individual level, for 4 out of those 5 patients for whom longitudinal SARA data were available, the disease progression rate was indeed more similar to the disease progression rate of the patients with non-genetic MSA-C_cp_ (Fig. 2a), than that of the GAA-*FGF14* SAOA cohort (i.e., SARA disease progression rate in 4/5 GAA-*FGF14* MSA-C_cp_ subjects >2 SARA points/year: Patient 3 (P3): 4 points/year; P5: 3.7 points/year, P6: 2.7 points/year; P7: 2.1 points/year). This indicates that in some patients with GAA*-FGF14*-ataxia, the disease progression is similar to MSA-C—either as they are part of a more rapid disease cluster in GAA-*FGF14* ataxia or because they have MSA-C as a coexisting, second condition.

Cross-sectional comparison of NfL levels revealed that NfL levels in the GAA-*FGF14* MSA-C_cp_ group (12.9 pg/mL, IQR 11.6–14.4, n = 4) were significantly lower than in the non-genetic MSA-C_cp_ group (27.1 pg/mL, IQR 19.5–35.6, n = 58, p < 0.05) and more similar to the NfL levels of patients with GAA-*FGF14* SAOA (15.5 pg/mL, IQR 10.3–20.6, n = 15, p > 0.05–LMEM) (Fig. 2b). This indicates that, at least on the overall group level, the rate of underlying axonal degeneration in patients with GAA-*FGF14* expansions with the MSA-C_cp_ phenotype is substantially less than in patients with non-genetic MSA-C_cp_.^29^^,^^30^

Biallelic *FGF14* (GAA)_≥250_ repeat expansions were identified in one individual (patient with non-MSA-C_cp_ SAOA) (for further details on this subject, see Supplement 5). Of note, we also identified 16/377 (4.2%) individuals with potentially pathogenic intermediate *FGF14* (GAA)_200-249_ alleles in our cohort (Supplement 6), which presents an enrichment compared to controls (19/2191 (=0.87%)); (4.2% vs 0.87%; OR 5.06, 95% CI 2.41–10.50, Fisher's exact test p < 0.0001).

### *RFC1* repeat expansions in sporadic ataxia

Biallelic *RFC1* repeat expansions of the pathogenic AAGGG motif were identified in 17/377 (4.5%) individuals, thereof 14/229 (6.1%) patients in the SAOA cohort and 3/148 (2.0%) patients in the MSA-C_cp_ cohort (Supplement 7). This includes one SAOA patient (subject P77) in whom, in addition to the biallelic *RFC1* repeat expansions, a potentially pathogenic intermediate GAA-*FGF14* repeat expansion (236 GAA repeat units) was identified. Of the three patients with *RFC1* expansions with an MSA-C_cp_ phenotype, the MSA-C_cp_ criterion of autonomic dysfunction was met in 1/3 patients with orthostatic hypotension, and 2/3 patients with genitourinary dysfunction (Table 1, and for additional phenotypic information see Supplement 11). Age of onset was 47 years (IQR 46.5–48), current age was 61 years (IQR 61–62.5), and disease duration was 15.8 years (IQR 15.0–15.8) in the *RFC1* MSA-C_cp_ group (n = 3). Age of onset and current age were significantly lower in the *RFC1* MSA-C_cp_ group than in the *RFC1* SAOA group (n = 14; age of onset: 54.5 years [IQR 49.3–59.8], current age 67 years [IQR 60.5–72.8], disease duration 13.6 years [IQR 11.6–17.2]; compared to *RFC1* MSA-C_cp:_ p ≤ 0.01, <0.05, >0.05 respectively student's t-test). This indicates that it was not a potentially higher age and/or disease duration that might have contributed to the MSA_cp_-phenotype in those patients with *RFC1* meeting the MSA-C_cp_ criteria. All 3/3 subjects with *RFC1* MSA-C_cp_ showed, in addition to ataxia, clinical signs associated with CANVAS and demonstrative of sensory neuropathy/ganglionopathy (absent distal tendon reflexes, at least of the Achilles tendon reflex; plus reduced vibration sense in all 3/3 patients), vestibular impairment when assessed (impaired head impulse test in all 2/2 patients), plus chronic cough (3/3 patients) (Supplement 11). This indicates that in at least some subjects with *RFC1* MSA-C_cp_ the MSA-like phenotype may present a phenotypic cluster along the continuous phenotypic spectrum of *RFC1*-ataxia and CANVAS.

All three patients with MSA-C_cp_ and *RFC1* expansions had high levels of ataxia severity (SARA score) relative to time since onset already at their first assessments (Fig. 2c) (P10, 10 SARA points; P11, 9.5 SARA points; P12, 20 SARA points), which were closer to the range of non-genetic MSA-C_cp_ patients, rather than of SAOA patients with *RFC1* expansions (Fig. 2c). However, longitudinal disease progression in patients with *RFC1* MSA-C_cp_ (0.85 ± 0.71 points/year; n = 3) was substantially slower than disease progression of patients with non-genetic MSA-C_cp_ (2.15 ± 0.16 points/year; n = 72), and more similar to patients with *RFC1* SAOA (1.16 ± 0.36 points/year; n = 14). Comparisons between these groups for disease severity relative to time since onset at first assessment and longitudinal progression both did not reach statistical significance, likely due to the small patient numbers. Cross-sectional comparison of NfL levels showed that NfL levels in the *RFC1* MSA-C_cp_ group (21.9 pg/mL, IQR 16.0–27.7, n = 2) were lower than in the non-genetic MSA-C_cp_ group (27.1 pg/mL, IQR 19.5–35.6, n = 58) and more similar to *RFC1* SAOA NfL levels (18.4 pg/mL, IQR 15.5–24.6, n = 8), but also not reaching significance likely due to small patient numbers (*RFC1* MSA-C_cp_ n = 3 and *RFC1* SAOA n = 14 respectively). The *RFC1* SAOA group also showed no significant difference in NfL levels compared to the non-genetic MSA-C_cp_ group (p > 0.05 - LMEM) (Fig. 2d). Taken together, these results indicate that the MSA-like phenotype might represent a phenotypic cluster along the continuous phenotypic spectrum of *RFC1*-ataxia, with MSA-like symptoms and high ataxia severity already early in the disease course, but still without MSA-typical progression rates or MSA-typical rates of axonal degeneration.

### Single-nucleotide variants in sporadic ataxia

Next-generation sequencing was used to investigate causative SNVs in this cohort, yielding 22 definitive or probable genetic findings in 21/377 individuals (5.8%) in the total cohort (Fig. 1a–c, Table 2, Table 3), thereof 14/229 (6.1%) in the SAOA group and 6/148 (4.1%) in the MSA-C_cp_ group. Unclear genetic findings were identified in 3 of 377 (0.8%) patients, in the form of pathogenic or likely pathogenic variants for which the contribution to the ataxia phenotype remains unclear, were identified in the total cohort, thereof 3/229 (1.3%) in the SAOA group (Fig. 1b and c). In total, 24/377 (6.4%) patients had SNVs identified by NGS in the definitive, probable, unclear categories (Fig. 1b and c). From a testing strategy perspective, the diagnostic yield for SNVs in whole exome sequencing (WES)-tested individuals (10.8%) was overall higher than those subjected to targeted gene panel sequencing (6.4%) (Supplement 9).

**Table 2:** Autosomal-dominant genetic findings by next-generation sequencing.

Individual#	Sporadic ataxia cluster	Clinical features	Gene	OMIM	Zygosity	Variant (hg19)	GnomADv2	CADD (GRCh37-v1.7)	SIFT	PolyPhen2	ACMG classification	Genetic diagnosis for causing the ataxia phenotype
**Definitive and probable genetic diagnoses**												

P19	SAOA	Ataxia with ATR areflexia, moderate Impaired vibration sense (LL) and hypermetric saccades	*CACNA1A*	601011	heterozygous	NM_001127222.2: c.3882+2T > C (p.?)	0.00E+00	16.3	NA	NA	Pathogenic (PVS1, PM2, PP3)	Definitive
P20	SAOA	Ataxia with mild LL paresis, broken up smooth pursuit, mild dysphagia.	*CACNA1A*	601011	heterozygous	NM_001126131.2: c.1987C > T (p.Gln663Ter)	0.00E+00	52.0	NA	NA	Likely Pathogenic (PVS1, PM2, PP3, PP5)	Definitive
P13	MSA-C_cp_	Ataxia with bilateral positive Babinski reflex, slowing of saccades, mild urinary dysfunction.	*CACNA1A*	601011	heterozygous	NM_001127222.2: c.2404C > A (p.Arg802Ser)	4.72E-06	25.9	Deleterious	Possibly damaging	Likely Pathogenic (PM1, PM2, PP2, PP3, PP4)	Definitive
P21	SAOA	Ataxia with mild LL paresis, broken up smooth pursuit, gaze-evoked nystagmus, slowed saccades, strabismus.	*CACNA1A*	601011	heterozygous	NM_001127222.2: c.593G > A (p.Arg198Gln)	0.00E+00	28.6	Deleterious	Probably damaging	Likely Pathogenic (PM1, PM2, PP3)	Definitive
P14	MSA-C_cp_	Ataxia with ATR and PTR areflexia, mild limb spasticity, broken up smooth pursuit, dysphagia, and moderate urinary incontinence	*GFAP*	137780	heterozygous	NM_002055.5: c.208C > T (p.Arg70Trp)	0.00E+00	25.6	Deleterious	Probably damaging	Likely Pathogenic (PM1, PM2, PP3, PP5)	Definitive
P18	MSA-C_cp_	Ataxia with moderate Impaired vibration sense (LL), broken up smooth pursuit, hypermetric saccades, Impaired visual acuity, dysphagia, severe urinary incontinence, severe cognitive impairment	*KCNC3*	176264	heterozygous	NM_004977.3: c.1313G > A (p.Gly438Glu)	0.00E+00	27.7	Deleterious	Probably damaging	Likely Pathogenic (PM1, PM2, PP2, PP3)	Definitive
P15	MSA-C_cp_	Ataxia with PTR hyperreflexia, limb spasticity, gait spasticity, moderate Impaired vibration sense (LL), broken up smooth pursuit, horizontal nystagmus, slowed saccades, Impaired visual acuity, dysphagia, moderate urinary incontinence, mild cognitive impairment, sleep apnoea	*SLC1A3*	600111	heterozygous	NM_004172.5: c.1099G > A (p.Ala367Thr)	0.00E+00	22.2	Deleterious	Probably damaging	Likely Pathogenic (PM1, PM2, PP3, PP4)	Definitive
P22	SAOA	Ataxia with neck, truncal and UL chorea	*STUB1*	607207	heterozygous	NM_005861.4: c.613-1G > T (p.?)	0.00E+00	13.7	NA	NA	Pathogenic (PVS1, PM2, PP3)	Definitive
P23	SAOA	Ataxia with UL and LL areflexia, mild distal muscle atrophy LL, severely impaired vibration sense (LL), broken up smooth pursuit, hypo- and hypersaccades, dysphagia and mild urinary dysfunction	*TMEM240*	616101	heterozygous	NM_001114748.2: c.239C > T (p.Thr80Met)	0.0000385	12.0	Deleterious	Probably damaging	Likely Pathogenic (PM1, PM2, PP2, PP5)	Definitive
P24	SAOA	Ataxia with LL hyperreflexia, moderate LL paresis, broken up smooth pursuit, hypometric saccades, gaze evoked nystagmus	*TRPC3*	602345	heterozygous	NM_001130698.2: c.2620G > T (p.Glu874Ter)	0.00E+00	48.0	NA	NA	Pathogenic (PVS1, PM2, PP3)	Definitive
P25	SAOA	Ataxia with ATR areflexia, moderate distal muscle atrophy LL, broken up smooth pursuit, gaze-evoked nystagmus, dysphagia, urinary dysfunction, and mild cognitive impairment	*CACNA1G*	604065	heterozygous	NM_018896.5: c.4855G > A (p.Gly1619Ser)	4.10E-06	22.4	Tolerated	Probably damaging	Likely Pathogenic (PM1, PM2, PP2, PP3)	Probable (also probable genetic diagnosis of *PEX26*)
P26	SAOA	Ataxia with mild loss of vibration sense in distal LL, dysphagia, and mild urinary dysfunction	*CSF1R*	164770	heterozygous	NM_005211.3: c.1447_1450dup (p.Glu484ValfsTer30)	0.00E+00	NA	NA	NA	Pathogenic (PVS1, PM2, PP3)	Probable
P27	SAOA	Ataxia with mild muscle atrophy UL and LL, mild loss of vibration sense in distal LL, broken up smooth pursuit, slowed saccades, hypometric saccades and dysphagia	*KCND3*	605411	heterozygous	NM_004980.5: c.1119G > A (p.Met373Ile)	0.00E+00	23.2	Tolerated	Benign	Likely Pathogenic (PM1, PM2, PP2, PP3)	Probable
**Unclear genetic findings**												

P28	SAOA	Mild distal LL paresis, broken up smooth pursuit, gaze evoked-nystagmus, hypermetric saccades, Impaired visual acuity.	*OPA1*	605290	heterozygous	NM_130837.3: c.1311A > G (p.Ile437Met)	6.22E-04	23.1	Deleterious	Probably damaging	Likely Pathogenic (PM1, PM2, PP2, PP3)	Unclear (lacks the characteristic optic atrophy associated with *OPA1*)
**Genetic findings not causative of the ataxia phenotype**												

P29	MSA-C_cp_	Ataxia with moderate Impaired vibration sense (LL), broken up smooth pursuit, hypometric and hypermetric saccades, and severe urinary dysfunction (catheter)	*CACNB4*	601949	heterozygous	NM_000726.5: c.311G > T (p.Cys104Phe)	0.0004984	25.9	Deleterious	Probably damaging	Likely Pathogenic (PM1, PM2, PP2, PP3)	No (possibly a risk factor)
P30	SAOA	Ataxia with severe resting tremor, mild loss of vibration sense in distal LL, broken up smooth pursuit and dysphagia	*MFN2*	608507	heterozygous	NM_001127660.1: c.227T > G (p.Leu76Arg)	0.00E+00	21.6	Tolerated	Benign	Pathogenic (PM1, PM2, PM5, PP3)	No (non-ataxia disease variant, no neuropathy in patient)
P31	SAOA	Ataxia with ATR and PTR areflexia, mild spastic gait, mild muscle atrophy UL and LL, moderate loss of vibration sense in distal LL, broken up smooth pursuit, slowed saccades, opthalmoparesis on vertical gaze and dysphagia	*SOD1*	147450	heterozygous	NM_000454.5: c.217G > A (p.Gly73Ser)	0.00E+00	29.1	Deleterious	Probably damaging	Pathogenic (PS1, PM1, PM2, PP3)	Unlikely (not responsible for the ataxia phenotype, also homozygous *RFC1* AAGGG expansion fully explaining the ataxia phenotype)
P32	SAOA	Ataxia with mild muscle atrophy UL and LL, mild loss of vibration sense in distal LL, broken up smooth pursuit, slowed saccades, hypometric saccades and mild dysphagia	*SOD1*	147450	heterozygous	NM_000454.5: c.160A > G (p.Asn54Asp)	0.00E+00	23.4	Deleterious	Benign	Likely Pathogenic (PM1, PMP2, PP2, PP3)	Unlikely (not responsible for the ataxia phenotype, as also GAA-*FGF14* expansion 294 length fully explaining the ataxia phenotype)

Abbreviations: #, number; ATR, achilles tendon reflex; CADD, Combined Annotation Dependent Depletion score; HGVS, Human Genome Variation Society; LL, lower limb; MSA-C_cp_, clinically probable multiple system atrophy cerebellar type; NA, not applicable; OMIM, Online Mendelian Inheritance in Man; PolyPhen2, polymorphism phenotyping v2 based on HumDiv; PTR, patellar tendon reflex; SAOA, sporadic adult-onset ataxia of unknown aetiology; SIFT, scale-invariant feature transform (algorithm to predict the effects of coding non-synonymous variants on protein function); UL, upper limb.

**Table 3:** Autosomal-recessive genetic findings by next-generation sequencing.

Individual#	Phenotype	Clinical comments	Gene	OMIM	Zygosity	Variant (hg38)	GnomADv2	CADD (GRCh37-v1.7))	SIFT	PolyPhen2	ACMG classification	Genetic diagnosis for causing the ataxia phenotype
**Definitive and probable genetic diagnoses**												

P33	SAOA	Ataxia with mild paresis UL proximal, broken up smooth pursuit.	*ADCK3/COQ8A*	606980	heterozygous	NM_020247.5: c.1012G > A (p.Ala338Thr)	8.68E-06	25.1	D	Probably damaging	VUS (PM1, PM2, PP3, PP6)	Definitive
					heterozygous	NM_020247.5: c.1750_1752del (p.Thr584del)	9.99E-05	NA	NA	NA	Likely Pathogenic (PM1, PM2, PM4, PP6)	
P34	SAOA	Ataxia with UL and LL areflexia, moderate Impaired vibration sense (LL), moderate distal muscle atrophy LL, broken up smooth pursuit, gaze-evoked nystagmus, and hypermetric saccades	*ATM*	607585	heterozygous	NM_000051.4: c.4882A > G (p.Met1628Val)	0	22.3	D	Benign	Likely Pathogenic (PM1, PM2, PP2, PP3, PP6)	Definitive
					heterozygous	NM_000051.4:c.8264A > C (p.Tyr2755Ser)	0	19.6	D	Probably damaging	Pathogenic (PM1, PM2, PP2, PP3, PP4, PP6)	
P16	MSA-C_cp_	Ataxia with hyperreflexia (biceps, patella, Achilles), mildly Impaired vibration sense (LL), moderate dysphagia, moderate urinary dysfunction, bradykinesia,	*ATM*	607585	heterozygous	NM_000051.4: c.94C > T (p.Arg32Cys)	3.19E-05	26.5	D	Possibly damaging	VUS (PM2, PP2, PP3, PP4, PP6)	Definitive
					heterozygous	NM_000051.4: c.1339C > T (p.Arg447Ter)	0.00E+00	37.0	NA	NA	Likely Pathogenic (PVS1, PM2, PP3, PP4, PP6)	
P35	SAOA	Ataxia with mild resting tremor	*SNX14*	616105	heterozygous	NM_153816.6: c.2670del (p.Cys890Ter)	6.39E-05	NA	NA	NA	Pathogenic (PVS1, PM2, PP3)	Definitive
					heterozygous	NM_153816.6: c.512G > A (p.Arg171His)	0.00E+00	26.9	T	Probably damaging	VUS (PM1, PM2, PP3, BP1)	
P36	SAOA	Ataxia with PTR hyperreflexia, mild spasticity UL, mild LL paresis, broken up smooth pursuit, ophthalmoparesis, slowed and hypometric saccades, mild cognitive impairment, erectile dysfunction	*SPG7*	602783	homozygous	NM_003119.4: c.1529C > T (p.Ala510Val)	2.92E-03	29.2	D	Probably damaging	Pathogenic (PS3, PS4, PM1, PM2, PP3)	Definitive
P37	SAOA	Ataxia with mild distal LL paresis, hypometric saccades, mild dysphagia, erectile dysfunction	*SPG7*	602783	homozygous	NM_003119.4: c.1045G > A (p.Gly349Ser)	8.34E-04	26.4	D	Probably damaging	Likely Pathogenic (PM1, PM2, PP2, PP3, PP5)	Definitive
P38	SAOA	Ataxia with LL areflexia and bilateral positive Babinski sign, spastic gait, limb spasticity (LL), moderate Impaired vibration sense (LL), slowed saccades, horizontal and vertical opthalmoparesis, dysphagia and urinary dysfunction	*SPG7*	602783	heterozygous	NM_003119.4: c.1529C > T (p.Ala510Val)	2.92E-03	29.2	D	Probably damaging	Pathogenic (PS3, PS4, PM1, PM2, PP3, PP6)	Definitive
					heterozygous	NM_003119.4: c.2161A > G (p.Asn721Asp)	0	26.9	D	Probably damaging	Pathogenic (PM1, PM2, PP2, PP3, PP6)	
P17	MSA-C_cp_	Ataxia with ATR and PTR areflexia, spastic gait, LL spasticity, broken up smooth pursuit, horizontal and vertical opthalmoparesis and urinary dysfunction	*SPG7*	602783	heterozygous	NM_003119.4: c.1529C > T (p.Ala510Val)	2.92E-03	29.2	D	Probably damaging	Pathogenic (PS3, PS4, PM1, PM2, PP3, PP6)	Definitive
					heterozygous	NM_003119.4:c.1552+1G > T (p.?)	0.0000203	25.4	NA	NA	Pathogenic (PVS1, PM2, PP3, PP6)	
P25	SAOA	Ataxia with ATR areflexia, moderate distal muscle atrophy LL, broken up smooth pursuit, gaze-evoked nystagmus, dysphagia, urinary dysfunction, and mild cognitive impairment	*PEX26*	608666	heterozygous	NM_017929.6: c.200A > G (p.Asn67Ser)	1.40E-03	8.9	T	Benign	VUS (PM1, PM2, PP6, BP4)	Probable (also probable genetic diagnosis of *CACNA1G*)
					heterozygous	NM_017929.6: c.292C > T (p.Arg98Trp)	6.49E-05	22.6	D	Probably damaging	Likely Pathogenic (PM1, PM2, PP3, PP5, PP6)	
**Unclear genetic findings**												

P39	SAOA	Ataxia with mild rigidity (axial + UL + LL), moderate Impaired vibration sense (LL), broken up smooth pursuit, square wave jerks on fixation, downbeat- and gaze evoked nystagmus, slowed and hypometric and hypermetric saccades, moderate urinary dysfunction	*POLG*	174763	heterozygous	NM_001126131.2: c.1760C > T (p.Pro587Leu)	1.49E-03	24.5	D	Probably damaging	Pathogenic (PS1, PS3, PM2, PP3, BP2)	Unclear (not proven in trans, commonly in cis)
					heterozygous	NM_001126131.2: c.752C > T (p.Thr251Ile)	1.55E-03	13.5	T	Benign	Pathogenic (PS1, PS3, PM2, PP3, BP2)	
P40	SAOA	Ataxia with ATR areflexia, mild UL muscle atrophy, UL muscle myoclonus, UL rigidity, mild resting tremor, moderately impaired vibration sense (LL), broken up smooth pursuit, downbeat nystagmus, gaze evoked horizontal nystagmus, hypersaccades, impaired visual acuity, moderate dysphagia, and moderate urinary dysfunction	*POLG*	174763	heterozygous	NM_001126131.2: c.1760C > T (p.Pro587Leu)	1.49E-03	24.5	D	Probably damaging	Pathogenic (PS1, PS3, PM2, PP3, BP2)	Unclear (not proven in trans, commonly in cis, GAA-*FGF14* expansion 274 length)
					heterozygous	NM_001126131.2: c.752C > T (p.Thr251Ile)	1.55E-03	13.5	T	Benign	Pathogenic (PS1, PS3, PM2, PP3, BP2)	
					heterozygous	NM_003119.4: c.1552+1G > T (p.?) (p.?)	0.0000203	25.4	NA	NA	Pathogenic (PVS1, PM2, PP3, PP6)	

Abbreviations: #, number; ATR, achilles tendon reflex; CADD, Combined Annotation Dependent Depletion score; D, deleterious; HGVS, Human Genome Variation Society; MSA-C_cp_, clinically probable multiple system atrophy cerebellar type; NA, not applicable; OMIM, Online Mendelian Inheritance in Man; PolyPhen2, polymorphism phenotyping v2 based on HumDiv; PTR, patellar tendon reflex; SAOA, sporadic adult-onset ataxia of unknown aetiology; SIFT, scale-invariant feature transform (algorithm to predict the effects of coding non-synonymous variants on protein function); T, tolerated.

Causative SNVs were identified in both autosomal-dominant and autosomal-recessive genes for both phenotypic subgroups (Fig. 1b and c). *CACNA1A* (n = 4) and *SPG7* (n = 4) were the most common genetic causes, with these genes accounting for four genetic diagnoses each. Variants in *ATM* (n = 2) were causative for the ataxia phenotype in two cases. All other genes (n = 11) accounted for a single definitive or probable genetic diagnosis each: *ADCK3/COQ8A*, *CACNA1G, CSF1R, GFAP*, *KCNC3*, *PEX26*, *SLC1A3*, *SNX14*, *STUB1*, *TRPC3*, *TMEM240* (Tables 2 and 3). Out of these genes, pathogenic variants were found in the MSA-C_cp_ group in the following genes: *ATM*, *CACNA1A*, *GFAP*, *KCNC3*, *SLC1A3* and *SPG7* (Tables 2 and 3, Fig. 1b and c). All 6/6 of these patients with MSA-C_cp_ phenotype met the MSA criterion of autonomic dysfunction due to genitourinary dysfunction, while they did not show orthostatic hypotension (Table 1). The subject carrying the *ATM* variants (patient P16) also showed all MRI signs characteristic of MSA on imaging (hot cross bun sign, atrophy of pons and middle cerebellar peduncles), thus making it likely that she indeed had MSA-C plus a genetic condition (Ataxia Telangiectasia caused by mutations in *ATM*) as two independent, co-occurring conditions (for case description, see Supplement 8).

Variants of unclear significance for the ataxia phenotype were identified in three cases: *OPA1* (n = 1), *POLG* (n = 2). P28 carries the likely pathogenic *OPA1* variant, however, lacks the characteristic optic atrophy phenotype. For individuals P39 and P40, variant phasing could not be established for the two pathogenic *POLG* heterozygous variants, and P40 also carries a pathogenic GAA-*FGF14* repeat expansion, thus potentially presenting a dual diagnosis.

Two patients had variants in *SOD1* (n = 2; P31, P32), both with a second, independent mutation in an ataxia gene, thus demonstrating a dual genetic diagnosis. Individual P31 carried a known pathogenic *SOD1* variant (c.217G > A, p.Gly73Ser)^31^), together with biallelic pathogenic *RFC1* repeat expansions, and in fact showed characteristic features of both an ataxia and a motor neuron disease (Table 2; for case description, see Supplement 8), indicating that the *SOD1* variant is likely not responsible for the ataxia phenotype. Individual P32 carried the likely pathogenic *SOD1* variant (c.160A > G (p.Asn54Asp)), together with a pathogenic GAA-*FGF14* repeat expansion (294 GAA repeat units). He showed no symptoms of a motor neuron disease, but showed a clear ataxia phenotype, including the classic clinical characteristics of SCA27B e.g., severe downbeat nystagmus. This does, of course, not exclude a potential pathogenicity of this *SOD1* variant, e.g., as a dual genetic diagnosis in an as of yet asymptomatic patient (for case description, see Supplement 8). Similarly, a known pathogenic *MFN2* c.227T > G (p.Leu76Arg) mutation, causative for Charcot-Marie-Tooth disease, was identified; however, the patient displayed no clear peripheral neuropathy phenotype and isolated adult-onset ataxia is not a phenotype currently associated with *MFN2* mutations. These findings indicate that a certain share of patients with sporadic degenerative ataxia (at least 3/377 = 0.8%) can carry pathogenic mutations in other, non-ataxia known neurological monogenetic disease genes (e.g., *SOD1*, *MFN2*), but where they likely do not explain the actual manifest neurological disease of ataxia, yet present a genetic risk for a second, non-manifest neurological disease.

### Dual genetic diagnoses

Overall, one patient was found to carry pathogenic variants in two different ataxia genes (*CACNA1G* + *PEX26*); two patients had one pathogenic variant in one ataxia gene plus one possibly pathogenic variant in another ataxia gene (*RFC1* + GAA-*FGF14*_236_ and GAA-*FGF14*_274_ + *POLG* mutations); and two patients had pathogenic variants in one ataxia gene plus one other rare neurological disease gene (GAA-*FGF14*_294_ + *SOD1*_N54D_ mutation, *RFC1* + *SOD1*_G73S_). The overall frequency of dual genetic diagnosis in this cohort was 5/377 = 1.3% (Fig. 1a).

## Discussion

Here we provide a systematic assessment of the genetic landscape of sporadic adult-onset ataxia, combining multi-modal genetic screening techniques (NGS plus repeat sequencing techniques, including optimised techniques for recent genes such as GAA*-FGF14*) in a well-characterised prospective large multi-centre cohort (SPORTAX), combined with longitudinal natural history data. This allowed us to provide not only estimates on relative frequencies in sporadic adult-onset ataxia; but also insights on variant types, dual genetic diagnoses, and genetic findings in patients with ataxia meeting the, now revised,^22^ 2nd consensus MSA_cp_ diagnostic criteria.

### The genetic landscape of sporadic (late-) adult-onset ataxia

Our findings demonstrate a substantial burden of monogenic causes in sporadic adult-onset ataxia, with 22.5% (85/377) patients carrying a pathogenic or likely pathogenic variant, thereof 67/229 (29.3%) patients with SAOA and even 18/148 (12.2%) patients meeting the MSA-C_cp_ criteria. This frequency is a substantially conservative estimate as the most common SCA CAG-repeat expansions, which account for up to 10–15% of sporadic adult-onset ataxia in non pre-stratified cohorts,^1^^,^^2^ had to be excluded prior to inclusion per SPORTAX study cohort enrolment criteria. This high overall frequency of monogenic causes in sporadic adult-onset ataxia is somewhat surprising not only compared to most other sporadic adult-onset neurodegenerative diseases including Alzheimer's disease, amyotrophic lateral sclerosis, or Parkinson's disease (which only have a relatively minor monogenic component of 5–10%^32^, ^33^, ^34^, ^35^, ^36^, ^37^), but also from an ataxia perspective. Albeit genetic studies in other ataxia cohorts have rendered similar frequencies in the range of 20–30% monogenic causes,^38^, ^39^, ^40^ these screening studies—unlike SPORTAX—have not focused strictly on sporadic (late) adult-onset ataxia. SPORTAX included only (i) *late* adult-onset patients (age of onset >40 years), with (ii) negative family history and (iii) exclusion of the common CAG-repeat SCAs (see inclusion criteria), thus making them less likely to have a genetic cause. Applying these criteria would likely have led to substantially smaller frequencies in other prior studies.

Our study indicates that high monogenic burden of sporadic adult-onset ataxia is due to substantial contributions of repeat expansions. Here, 72.9% (62/85) patients with a genetic diagnosis carried disease-causing repeat expansions in one of these two genes (GAA-*FGF14*_≥250_ 45/85 (52.9%) and *RFC1* 17/85 (20.0%)), thus confirming their role as key loci of adult-onset ataxia, now even in patients with sporadic ataxia. This might also explain why, compared to previous studies, our study was able to unravel such a high monogenic contribution in sporadic adult-onset ataxia. Many other previous cohort studies^38^^,^^40^^,^^41^ had not been able to test for these two loci as they were only quite recently identified in 2019 and 2022 respectively, in particular GAA-*FGF14* (SCA27B).^4^^,^^5^

Patients with pathogenic and likely pathogenic SNVs also account for a relevant share of sporadic adult-onset ataxia (21/377, 5.6%). These are highly heterogeneous with 16 genes contributing, and only three genes presenting a recurrent cause (i.e., >1 patient). The genes that account for more than one genetic diagnosis in our cohort were *ATM*, *CACNA1A* and *SPG7*, three genes that are also very frequent genes in the broader spectrum of ataxia disorders including early-onset and familial types.^38^^,^^42^^,^^43^

### GAA*-FGF14* as the main genetic contributor to sporadic adult-onset ataxia

Together with the *RFC1* expansion, GAA*-FGF14* repeat expansions were the main genetic contributor of sporadic adult-onset ataxia in our cohort. A total of 45 patients with GAA-*FGF14*_≥250_ were identified, accounting for 52.9% (45/85) of all patients with sporadic ataxia where a genetic diagnosis could be established, and 11.9% (45/377) of the total group. The identification of such a high frequency of patients with a variant in an autosomal-dominant disease gene might be surprising at first glance in a patient cohort of sporadic disease, where patients with similar disorders in first- and second-degree relatives had been explicitly excluded upfront by study design. Yet, this finding is well in line with other recent studies, reporting a sporadic presentation of GAA-*FGF14* disease in 15–50% patients,^13^^,^^44^, ^45^, ^46^, ^47^ with our results thus further corroborating the concept of an age-dependent reduced penetrance of GAA-*FGF14* repeat expansions.^4^^,^^48^^,^^49^

The finding of a high contribution of GAA-*FGF14* to sporadic adult-onset ataxia is important not only because of the high frequency, but importantly also given the direct actionable treatment implications. While sporadic ataxia without any identified genetic cause is currently not amenable to targeted drug treatments, an increasing body of evidence demonstrates that GAA-*FGF14* disease is readily treatable with 4-aminopyrdine (4-AP). In recent studies, up to 80% of patients with GAA-*FGF14* showed a clinician-reported treatment response to 4-AP,^19^ often of marked intraindividual effect size,^6^^,^^19^ and now documented by digital-motor recordings of both gait and nystagmus.^19^^,^^49^

In addition to the 45 patients with GAA-*FGF14*_≥250_, we also identified 16 individuals with GAA repeat expansions in the range of 200–250 GAA-*FGF14* repeat units, which presented a significant enrichment compared to controls.^19^^,^^50^^,^^51^ This finding substantiates the recent suggestion that the pathogenic threshold of GAA-*FGF14* disease might be lower than the previously established cutoff of 250 repeat units.^52^ Here we add indications that *FGF14*-GAA repeats of 200–249 repeat units could possibly be associated with an adult-onset cerebellar ataxia phenotype. Hypothetically, this ‘intermediate’ repeat range may in particular even be associated with a *sporadic* adult-onset cerebellar ataxia phenotype, as intermediate alleles with reduced penetrance are fitting with a sporadic appearance of disease inheritance. Future studies are warranted to test this hypothesis further.

### Genetic variants associated with MSA-C_cp_ phenotypes and clinico-molecular insights

Apart from 29.3% (67/229) patients with SAOA carrying a pathogenic or likely pathogenic variant, 12.2% (18/148) patients meeting the 2nd consensus MSA_cp_ diagnostic criteria (‘patients with MSA-C_cp_’) carried a pathogenic or likely pathogenic variant. This group likely comprises both, patients with ‘true’ MSA-C and patients with ataxia with MSA-C mimic phenotypes. It included nine patients with GAA-*FGF14*_≥250_, three with *RFC1* repeat expansions, and six with SNVs.

Our multi-modal in-depth assessments combining phenotypic features with longitudinal progression modelling and molecular biomarker (NfL) data indicated that the MSA-C_cp_ patients with GAA-*FGF14* expansions did not only meet the phenotypic criteria of MSA-C_cp_, but many of them also had a similar progression rate as non-genetic MSA-C_cp_ patients; while their plasma NfL levels were more similar to the patients with SAOA and GAA-*FGF14*. This indicates that in at least some patients with GAA-*FGF14* expansions, the MSA-like phenotype might represent a fast progression phenotypic cluster along the continuous phenotypic spectrum of GAA*-FGF14*-ataxia, while the rate of underlying axonal degeneration is substantially less than in patients with non-genetic MSA-C_cp_. Higher NfL levels in non-genetic MSA-C_cp_ may derive from a more widespread degeneration (e.g., of the brainstem) than in GAA*-FGF14*-ataxia.

The patients with MSA-C_cp_ and *RFC1* expansions showed high levels of ataxia severity relative to time since onset already at first assessments, in the same range as patients with non-genetic MSA-C_cp_. However, longitudinal disease progression rates and NfL levels were more similar to patients with SAOA and *RFC1* expansions than patients with non-genetic MSA-C_cp_. This indicates that the MSA-like phenotype may also represent a more severe phenotypic cluster along the continuous phenotypic spectrum of *RFC1* disease, starting early and more severe in the disease course of *RFC1* disease; yet it does still not show MSA-typical progression rates and MSA-typical axonal degeneration rates.

Our findings on the MSA-C_cp_ phenotypes in these respective genetic diseases not only corroborate, but substantially extend recent single case or small case series on the MSA-C_cp-_ phenotypes in GAA*-FGF14*^53^ and *RFC1* disease,^8^^,^^12^ respectively. They provide comparative insights into the longitudinal progression and underlying axonal degeneration rates of these MSA-C_cp_ phenotypes in GAA*-FGF14* and *RFC1* disease. Comparisons are provided to both a non-genetic MSA-C_cp_ cohort on the one hand and a non-MSA cohort of the respective genes on the other.

It is unlikely that any of the variants identified here in MSA-C_cp_ patients (including the SNVs) present a direct monogenic cause of definite MSA, i.e., an oligodendrocyte synucleinopathy. This would be mechanistically unlikely, and as shown here, the substantially slower axonal degeneration rate speaks against this possible explanation. The most conservative interpretation of these findings is that the group of MSA-C_cp_ patients comprises a substantial share of MSA-C mimics. MSA-C mimics are well known for other genetic ataxias like SCA1, SCA2, SCA3 or FXTAS.^54^ *RFC1*-ataxia and SCA27B should now be added to this list. Taken together, this underlines a lack of specificity of the 2008 2nd consensus MSA-C_cp_ criteria against the background of genetic ataxias. Specifically, autonomic dysfunction -in particular urinary incontinence and orthostatic hypotension-is a recurrent feature,^55^^,^^56^ (as shown here for patients with GAA-*FGF14* expansions, *RFC1* expansions and SNVs). Moreover, some patients with genetic ataxia even show MSA-like progression rates (GAA-*FGF14*) or MSA-like early severity levels (*RFC1*).

In addition, our MSA-C_cp_ group might also have included a certain, likely smaller share of ‘true’ MSA-C patients. Here some MSA-C_cp_ patients may carry pathogenic variants in ataxia genes just as a statistical coincidence (dual diagnosis), but not as the causally main driver of an actual MSA-C disease. This might be particularly true for GAA-*FGF14* and *RFC1* repeat expansion carriers, based on their high background frequency in the general population,^4^^,^^7^ and as suggested here by MSA-C_cp_ patients with GAA-*FGF14* expansions P6 and P7 who showed additional clinical signs highly characteristic for MSA-C; as well as likely for patient P16 with the *ATM* variants plus MSA-C_cp._

### Pathogenic variants unrelated to the ataxia phenotype

One patient (1/377 = 0.3%) where no primary genetic cause for the ataxia disease had been identified, carried a pathogenic mutation in another non-ataxia known neurological monogenetic disease gene (*MFN2*). This variant likely does not explain the actual ataxia phenotype, yet presents a genetic risk for a second non-manifest neurological disease. This illustrates the scrutiny required in the neurogenetics field to not prematurely claim a pathogenic contribution of a respective non-ataxia gene to the main disease phenotype. Rather, the more likely concept needs that to be explicitly appreciated is that patients can also carry pathogenic variants in not-yet-manifest disease genes, completely independent of the main manifest disease phenotype—which was recently similarly shown for ALS genetics.^57^

### Dual genetic diagnoses

In addition, 5/377 patients (1.3%) were found to have two relevant genetic findings simultaneously (dual genetic diagnosis). This finding corroborates and extends previous studies where 4%–6% of children diagnosed with a genetic disease also have a second, independent genetic diagnosis.^58^^,^^59^ We now demonstrate that even in one of the patient strata least likely to have a genetic cause—late adult-onset sporadic disease—dual genetic diagnoses are also a recurrent finding, even if considering only *neurological* disease genes. This finding is of immediate clinico-genetic diagnostic importance, as it highlights the need to consider genetic investigations for a second genetic diagnosis—and potentially also exclusion thereof—even in late adult-onset disease patients.

Yet with a quickly increasing number of targeted treatments for genetic diseases reaching clinical care, this finding is of direct treatment-relevance. For example, with GAA-*FGF14* ataxia now showing a substantial treatment-response to 4-aminopyridine,^6^^,^^19^ the corresponding treatment effect might be confounded—both in terms of efficacy as well as side effects—by the concurrent *SOD1* mutation in patients with sporadic adult-onset ataxia (as identified in P31 and P32). Additionally, in turn, with *SOD1* disease now being treatable by the antisense oligonucleotide Tofersen,^60^ any treatment effect from Tofersen might be confounded—both in terms of efficacy and by side effects—by the concurrent *RFC1* and GAA-*FGF14* mutations, respectively, in these two patients with ataxia.

### Strengths and limitations

Our study has several key strengths. It presents a systematic clinico-genetic analysis of a large, multi-centre, prospective cohort focused exclusively on sporadic adult-onset ataxia. Using comprehensive multi-modal genetic screening approach, including optimised methods for recently identified genes like GAA-*FGF14*, we were able to uncover a substantial monogenic burden in sporadic adult-onset ataxia - a cohort designed to be less likely to have a genetic cause. This even included a substantial share of patients meeting the MSA-C_cp_ criteria, suggesting a reduced specificity of this clinical diagnosis; and potential co-occurrence of MSA-C plus a second, independent genetic condition. Furthermore, our findings have direct clinical relevance, highlighting the importance of genetic testing in sporadic ataxia - even when presenting with MSA-like features–and paving the way for providing these patients with potential access to targeted treatments.

However, the results of our study need to be interpreted in light of some limitations. First, independent cohorts ideally from different ethnic backgrounds are needed to compare and replicate our findings. Second, while presenting a large number of patients with an MSA-C_cp_ phenotype associated with an underlying monogenic variant and in particular providing comparative insights into the longitudinal progression rates and underlying axonal turnover of these patients, still larger cohorts of MSA-C_cp_ patients with associated monogenic variants are required to study these phenotypic disease clusters in more depth. In particular, future studies are warranted to confirm the current findings on the GAA*-FGF14* and *RFC1* MSA-C_cp_ severity, progression and NfL levels, which are still preliminary in nature given the small sample sizes (e.g., n = 9 MSA-C_cp_ patients with GAA-*FGF14* expansions; n = 3 MSA-C_cp_ patients with *RFC1* expansions), Third, larger studies in definite (i.e., neuropathologically confirmed) MSA cases are highly warranted to allow disentangling in which MSA-C_cp_ patients the respective genetic variants are coincident second hits to an independent ‘true’ MSA pathology versus causes of an MSA-C-like phenotype without any MSA pathology. Similarly, future studies are also needed to prospectively characterise these MSA phenotypes according to the recent Movement Disorder Society MSA criteria,^22^ as these criteria might allow for improved specificity also against the background of genetic ataxias. Fourth, similar to previous studies,^19^ our findings of potential pathogenic contribution of *FGF14* GAA_200-249_ alleles are still preliminary and require validation through additional segregation studies, larger case–control series, and functional studies. Fifth, not all individuals for whom DNA was available were subjected to all available tests. NGS testing was not performed for 20.2% of these individuals (for GAA-*FGF14* and *RFC1* this was 5.6% and 4.5%, respectively). Based on these proportions of missing testing, we could expect that we are underestimating the relevant SNV findings that come from NGS when compared to the repeat expansion results. Sixth, future studies should complement the current genetic screening methods by methods for additional repeat motifs—e.g., especially additional *RFC1* repeat motifs^61^—and in particular by whole genome sequencing (given the large number of repeat expansions in sporadic ataxias) as the NGS data in our current study was not suitable to assess the presence of most repeat expansion loci. Ideally, additional repeat expansion loci would be tested by long-range whole genome sequencing, thereby potentially increasing the genetic yield even further by 8–20%,^62^, ^63^, ^64^ to further support that the frequency findings presented here may present a rather conservative estimate of the actual monogenic contribution to sporadic adult-onset ataxia.

In conclusion, leveraging a multi-modal genetic sequencing approach plus a strictly consecutive, prospective, multi-centre cohort, we demonstrated that sporadic adult-onset ataxias–as such less likely to have a genetic cause–have a substantial burden of monogenic variants, particularly GAA-*FGF14* and *RFC1* repeat expansions. This even includes a substantial share of patients with sporadic adult-onset ataxia meeting the 2008 2nd consensus MSA-C_cp_ diagnostic criteria. These findings have important implications for genetic work-up and counselling of patients with sporadic adult-onset ataxia, even when presenting with MSA-like features. With targeted treatments now on the horizon for genetic ataxias, they are also key for providing these patients with potential access to targeted therapies. Heightened sensitivity to potential second genetic diseases is necessary even in patients with (late) adult-onset sporadic ataxia, which might alter the treatment response to targeted treatments.

## Contributors

DB and MS—and in subparts CW—conceived the project, designed, and supervised the research. DB and MS wrote the initial draft of the manuscript and have accessed and verified the underlying data. DM conducted parts of the statistical analyses and interpretation thereof. DB, CW, DP, TH, OR, CD, SZ, BB and FH conducted several genetic analysis of the samples. TKlock designed and supervised the overall SPORTAX study and cohort. DO, AT, JF, DT, SB, SV, TKlop, BvdW, GS, CK, IMW, ZF, LS, and–in smaller share also the other SPORTAX consortium researchers–have conducted clinical assessments of subjects who participated in this study and collected biomaterial samples. All authors contributed to writing and revising the manuscript. All authors read and approved the final version of the manuscript.

## Data sharing statement

Individual de-identified patient data may be shared at the request of any qualified investigator upon reasonable request. No consent for open sharing has been obtained.

## Declaration of interests

Danique Beijer, Demet Önder, Carlo Wilke, Andreas Traschütz, Stefan Vielhaber, Thomas Klopstock, Florian Harmuth, Claudia Dufke, Bernard Brais, Olaf Rieß, Tobias B. Haack, Stephan Züchner, David Pellerin have no conflicts to report.

David Mengel received funding from the Clinician Scientist program “PRECISE.net” funded by the Else Kröner-Fresenius-Stiftung, the Clinician Scientist program of the Medical Faculty Tübingen (459-0-0), Elite Program for Postdoctoral researchers of the Baden-Württemberg-Foundation (1.16101.21), Ministry of Science, Research and the Arts of the State of Baden-Württemberg and the ARSACS Foundation.

Jennifer Faber received funding from the National Ataxia Foundation (NAF), was funded within the Advanced Clinician Scientist Programme (ACCENT, funding code 01EO2107). The ACCENT Program is funded by the German Federal Ministry of Education and Research (BMBF) and was funded as a PI within iBehave by the Ministry of Culture and Science of the State of North Rhine-Westphalia, Germany. JF was also reimbursed for education lecture expenses (diagnostic work-up and treatment of ataxia disorders) for practicing neurologists organised by continuing education companies; namely for organising a symposium for ASKLEPIOS Fachklinikum Stadtroda, and RG Gesellschaft für Information und Organisation mbH and has received consultancy honoraria as a participant of the strategic advisory board of VICO therapeutics.

Dagmar Timmann is receiving funding from the German Research Foundation (DFG), European Union, Bernd Fink-Foundation and Mercur, all unrelated to the present manuscript. DT has received payment from University of Hamburg and support to attend meetings from German Research Foundation and Gordon Conference Cerebellum.

Sylvia Boesch has received consulting fees from Biogen/Reata as well as honoraria from Biogen and Ipsen paid to the institution. SB has also participated in advisory boards for Vico Therapeutics and Biogen. SB has further received reimbursement of travel fees from Ipsen, Biogen/Reata and the Movement Disorders Society.

Bart van de Warrenburg reports funding from Hersenstichting, ZonMw, The Netherlands organization for scientific Research, Christina Foundation, and consultancy honoraria from Biogen, Servier, VICO therapeutics, and Biohaven Pharmaceuticals, as well as royalties from BSL/Springer nature, and honoraria from Mov disord council Malaysia. BvdW has served on the Scientific advisory boards for Biogen and Vico Therapeutics. BvdW has further participated as chair or member of the board for MDS ataxia study group, Dutch ataxia guideline committee, Dutch ataxia patient society, Dutch HSP patient community and ERN-RND and received equipment from Brugling Fund, all unrelated to the present manuscript.

Gabriella Silvestri has served as board member for aiViPS (Associazione italiana Vivere la Paraparesi Spastica) and of ARSACS odv.

Christoph Kamm has received royalties from Elsevier (co-author book chapter), lecture honoraria from Ipsen and meeting/travel support from Ipsen and Merz. CK further participated in boards for Biogen, Ipsen and Roche, all unrelated to the submitted work.

Iselin Marie Wedding reports no conflict of interest.

Zofia Fleszar is supported by the MINT-Clinician Scientist program of the Medical Faculty Tübingen, funded by the Deutsche Forschungsgemeinschaft (DFG, German Research Foundation)—493,665,037. ZF has also received consultancy fees from Healthcare Manufaktur GmbH.

Ludger Schöls has received funding from Servier and UCB as well as consulting fees from Vico Therapeutics and Vigil Neuroscience, and payment for expert testimony for Alexion and Novartis.

Thomas Klockgether has received, in the last 24 months, consulting fees from Bristol-Myers Squibb (BMS), UCB and Arrowhead, all unrelated to the present manuscript. TK has also been involved in leadership of the Ataxia Global Initiative (AGI).

Matthis Synofzik has received funding from the Else Kröner Fresenius Stiftung via a Clinician Scientist Programme Grant, as well as consultancy honoraria from Ionis Pharmaceuticals, UCB Pharmaceuticals, Prevail Pharmaceuticals, Orphazyme Pharmaceuticals, Servier Pharmaceuticals, Reata Pharmaceuticals, GenOrph, AviadoBio, Biohaven, Solaxa, Zevra, and Lilly, all unrelated to the present manuscript.

The SPORTAX consortium is funded in part by institutional in-house funding from the German Center of Neurodegenerative Diseases (DZNE) for the contributing DZNE sites. Remaining contributions are from sites’ own funding.

## Appendix A Supplementary data

SPORTAX_supplementary

Supplement

SPORTAX consortium author list
