# Supplementary material for: The genetic landscape of sporadic adult-onset degenerative ataxia: a multi-modal genetic study of 377 consecutive patients from the longitudinal multi-centre SPORTAX cohort
Source: eBioMedicine. 2025 Apr 23;115:105715. doi: 10.1016/j.ebiom.2025.105715 (PMC12051541; doi:10.1016/j.ebiom.2025.105715)
Supplement: SPORTAX_supplementary [file mmc1.pdf]

SUPPLEMENTARY INFORMATION

SUPPLEMENT 1: Multi-modal genetic investigation of the SPORTAX cohort

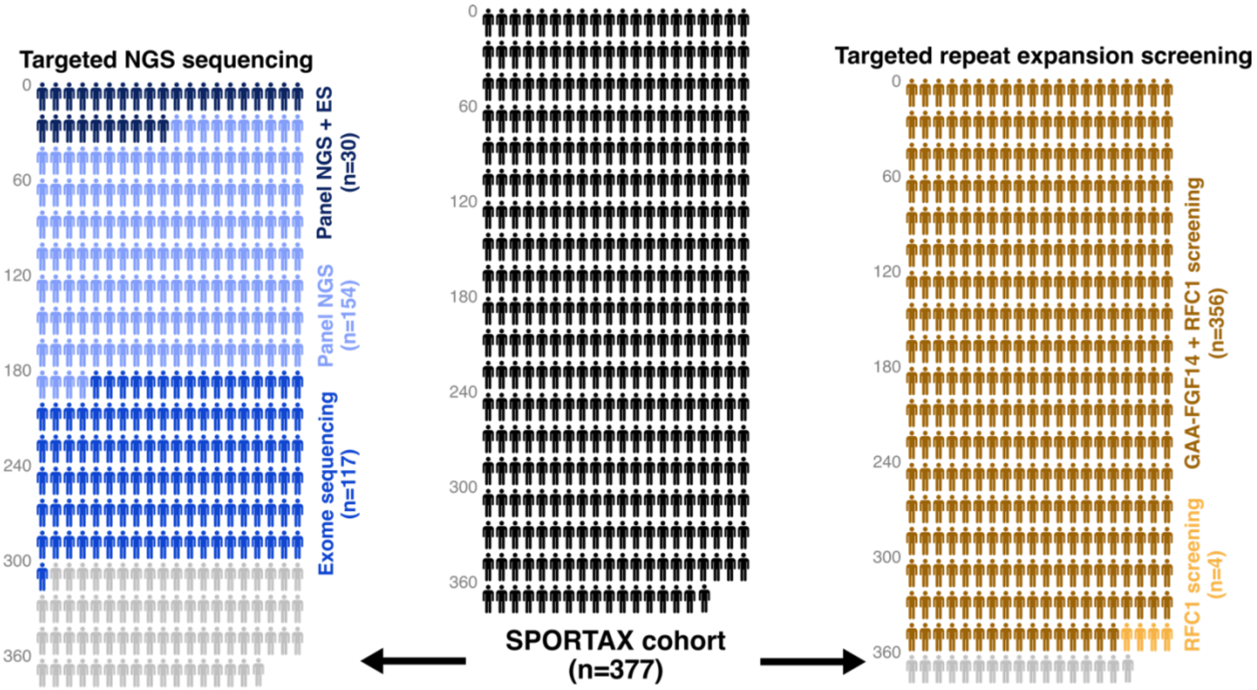

Supplement 1: 377 individuals from the SPORTAX registry were subjected to genetic testing. GAA-*FGF14* repeat expansion screening was available for 360 individuals, *RFC1* screening for 356 individuals next-generation sequencing (NGS) analyses of targeted gene-sets was performed for 301 individuals: dual-testing by panel NGS and exome sequencing (ES) for 30 individuals and exclusively panel NGS or ES for 154 and 117 individuals respectively.

SUPPLEMENT 2: Expanded methodology and gene sets for next-generation sequencing

Bioinformatic analysis of NGS data.

Called variants were subsequently filtered for read quality, read depth, population frequency, and for variant effect (frameshift, nonsense, splicing, or missense variants). For panel sequencing the process was described previously (1). For ES variant analysis, all variants called in the set of ataxia core genes (n=201) and ataxia-overlap disease genes (n=186) were separated into dominant and recessive inheritance-based filters (for dominant inheritance: heterozygous variants, Gnomad v2 allele frequency <0.001; for recessive inheritance: homozygous or (potentially) compound heterozygous variants GnomAD v2 allele frequency <0.01). Both dominant and recessive approaches were focused on variants with protein altering variant effects (frameshift, nonsense, missense, stop/start loss, in-frame deletion/insertion) or splice donor/acceptor sites. The following quality filters were employed: read depth >12, genotype quality >50 and quality filter >175. All variants were annotated with available mutation database information ClinVar to identify known rare variants with pathogenicity (2). Pathogenicity of the resulting variants was subsequently determined by applying ACMG criteria in a semi-automated way using InterVar with manual correction where needed and interpreted in the context of late-onset ataxia, resulting in a definitive, probable, unclear or no cause verdict of the variant's association with the observed phenotype (3-5). Manual correction of InterVar was performed as follows: (i) for PVS1 criteria, we manually verified their use to check the LoF as a known mechanism, in the correct inheritance pattern. (ii) For PM1 criteria, we also manually verified their use where applied to assess the accuracy of the hotspot. (iii) For PS3 criteria, we assessed the most recent literature to see if additional data on the effect of the mutation was published for these criteria, using a PubMed search for the gene and the single letter and three letter code of the mutation. (iv) For PP5 criteria, we also assessed ClinVar records when the variant was listed as "Pathogenic" or "Likely Pathogenic" and the submitters provided additional information that was not published elsewhere. For scoring of splice region variants, where our use of InterVar did not automate; these variants were thus scored manually, using the web version of InterVar.
VUS were not reported unless part of a pair of possible recessive variants or when the classification assessed in this study disagreed with prior assessment in ClinVar (2). For the analysis of the broad gene set of 976 genes linked with neurodegeneration we similarly assessed both dominant and recessive variants, irrespective of variant frequency, but filtered for variants with a Pathogenic or Likely Pathogenic classification in ClinVar (2).

Gene set #1: Core ataxia gene set (n = 201)

ABCB7, ABCD1, ABHD12, ACO2, ADCK3, AFG3L2, AHI1, ALAS2, ALG6, AMACR, AMT, ANO10, APTX, ARL13B, ARSA, ARX, ATCAY, ATM, ATP2B2, ATP2B3, ATP7B, B4GALNT1, BTD, C10ORF2, CA8, CACNA1A, CACNA1G, CACNB4, CAMTA1, CC2D2A, CEP290, CLN5, CLN6, CLN8, COQ2, COQ6, COQ8A, CP, CSTB, CYP27A1, CYP7B1, DARS, DARS2, DDB2, DLAT, DNAJC19, DNAJC3, DNAJC5, EEF2, EIF2B1, EIF2B2, EIF2B3, EIF2B4, EIF2B5, ELOVL4, ELOVL5, EPM2A, ERCC2, ERCC3, ERCC4, ERCC5, ETFA, ETFB, ETFDH, FGF14, FLVCR1, FOLR1, FXN, GALC, GBA, GBA2, GBE1, GCDH, GCLC, GCSH, GFAP, GLB1, GLDC, GM2A, GPR56, GRID2, GRM1, HERC1, HEXA, HEXB, HPRT1, HSD17B4, HTRA1, IGDCC3, INPP5E, ITM2B, ITPR1, KCNA1, KCNA2, KCNC1, KCNC3, KCND3, KCNJ10, KIAA0226, KIF1A, KIF1C, KIF5C, L2HGDH, MARS2, MFSD8, MLC1, MRE11A, MTPAP, MTRR, MTTP, NEU1, NHLRC1, NKX2-1, NOL3, NPC1, NPC2, NPHP1, OPA1, OPA3, PAX6, PDHX, PDSS1, PDSS2, PDYN, PEX10, PEX2, PEX7, PHYH, PIK3R5, PLA2G6, PLEKHG4, PMM2, PMPCA, PNKP, PNPLA6, POLG, POLH, POLR3A, POLR3B, PPT1, PRICKLE1, PRKCG, PRPS1, PRRT2, RAB3A, RARS2, RELN, RNF170, RNF216, RPGRIP1L, RRM2B, SACS, SCYL1, SEC16A, SETX, SIL1, SKOR1, SLC17A5, SLC19A3, SLC1A3, SLC25A15, SLC25A46, SLC2A1, SLC39A8, SLC52A2, SLC9A1, SNX14, SPG7, SPR, SPTBN2, STUB1, SUN1, SUN2, SYNE1, SYT14, TBCE, TDP1, TGM6, TMEM216, TMEM240, TMEM67, TPP1, TRPC3, TSEN2, TSEN34, TSEN54, TSFM, TTBK2, TTC19, TTPA, VAMP1, VLDLR, VPS13A, VRK1, VWA3B, WDR73, WFS1, WWOX, XPA, XPC, ZNF592

Gene set #2: Core ataxia + ataxia-overlap disease gene set (n = 387)

ABCB7, ABCD1, ABHD12, ACO2, ADCK3, AFG3L2, AHI1, AIMP1, ALAS2, ALDH18A1, ALDH3A2, ALG6, ALS2, AMACR, AMPD2, AMT, ANG, ANO10, AP4B1, AP4E1, AP4M1, AP4S1, AP5Z1, APTX, ARG1, ARL13B, ARL3, ARL6IP1, ARMC9, ARSA, ARSI, ARX, ATCAY, ATL1, ATM, ATP13A2, ATP1A3, ATP2B2, ATP2B3, ATP2B4, ATP7B, ATP8A2, ATXN8OS, AUH, B4GALNT1, B9D1, BEAN1, BICD2, BSCL2, BTD, C10ORF2, C12orf65, C19orf12, C5orf42, CA8, CACNA1A, CACNA1G, CACNB4, CAMTA1, CAPN1, CC2D2A, CCDC88C, CCT5, CEP104, CEP120, CEP290, CEP41, CLCN2, CLN5, CLN6, CLN8, COQ2, COQ6, COQ8A, CP, CPT1C, CSF1R, CSPP1, CSTB, CTDP1, CWF19L1, CYP27A1, CYP2U1, CYP7B1, DAB1, DARS, DARS2, DDB2, DDHD1, DDHD2, DLAT, DNAJC19, DNAJC3, DNAJC5, DNM2, DNMT1, DSTYK, EEF2, EIF2B1, EIF2B2, EIF2B3, EIF2B4, EIF2B5, ELOVL4, ELOVL5, ENTPD1, EPM2A, EPT1, ERCC2, ERCC3, ERCC4, ERCC5, ERLIN1, ERLIN2, ETFA, ETFB, ETFDH, EXOSC3, FA2H, FAM126A, FAM134B, FARS2, FBXO7, FGF14, FIG4, FLRT1, FLVCR1, FOLR1, FUS, FXN, GAD1, GALC, GAN, GARS1, GBA, GBA2, GBE1, GCDH, GCH1, GCLC, GCSH, GFAP, GJB1, GJC2, GLB1, GLDC, GLRX5, GLTP, GM2A, GNB1, GOSR2, GPR56, GPT2, GRID2, GRM1, HACE1, HEPACAM, HERC1, HEXA, HEXB, HPRT1, HSD17B4, HSPD1, HTRA1, HYLS1, IBA57, IFIH1, IFRD1, IGDCC3, INPP5E, INPP5K, IRF2BPL, ITM2B, ITPR1, KCNA1, KCNA2, KCNC1, KCNC3, KCND3, KCNJ10, KIAA0226, KIAA0556, KIAA0586, KIAA0753, KIDINS220, KIF1A, KIF1C, KIF5A, KIF5C, KIF7, KLC2, KLC4, KY, L1CAM, L2HGDH, LYST, MAG, MAN2B1, MARS1, MARS2, MFN2, MFSD8, MKS1, MLC1, MME, MRE11, MRE11A, MSTO1, MT-ATP6, MTPAP, MTRR, MTTP, NARS2, NDUFS7, NEU1, NHLRC1, NIPA1, NKX2-1, NKX6-2, NOL3, NPC1, NPC2, NPHP1, NT5C2, NUBPL, OFD1, OPA1, OPA3, OPTN, PANK2, PAX6, PCNA, PCYT2, PDE6D, PDHX, PDSS1, PDSS2, PDYN, PEX1, PEX10, PEX11B, PEX12, PEX13, PEX14, PEX16, PEX19, PEX2, PEX26, PEX3, PEX5, PEX6, PEX7, PGAP1, PHYH, PIBF1, PIK3R5, PLA2G6, PLEKHG4, PLP1, PMM2, PMPCA, PNKP, PNPLA6, POLG, POLH, POLR3A, POLR3B, PPT1, PRDX3, PRICKLE1, PRKCG, PRPS1, PRRT2, PTRH2, RAB3A, RAB3GAP2, RARS2, REEP1, REEP2, RELN, RNASEH2B, RNASET2, RNF168, RNF170, RNF216, RNU12, RPGRIP1L, RRM2B, RTN2, SACS, SCYL1, SEC16A, SERAC1, SETX, SIL1, SKOR1, SLC16A2, SLC17A5, SLC19A3, SLC1A3, SLC25A15, SLC25A46, SLC2A1, SLC33A1, SLC39A8, SLC52A2, SLC9A1, SLC9A6, SMPD1, SNAP25, SNX14, SOD1, SOX10, SPAST, SPG11,

SPG20, SPG21, SPG7, SPR, SPTAN1, SPTBN2, SQSTM1, STUB1, SUN1, SUN2, SYNE1, SYT14, TARDBP, TBCE, TCTN1, TCTN2, TCTN3, TDP1, TDP2, TECPR2, TFG, TGM6, TH, TMEM138, TMEM216, TMEM231, TMEM237, TMEM240, TMEM67, TPP1, TRAPPC11, TRPC3, TRPV4, TSEN2, TSEN34, TSEN54, TSFM, TTBK2, TTC19, TTPA, TUBB2B, TUBB4A, TWNK, UBAP1, UBQLN2, UBTF, USP8, VAMP1, VAPB, VARS2, VCP, VLDLR, VPS13A, VPS13D, VPS37A, VPS53, VRK1, VWA3B, WASHC5, WDR45, WDR48, WDR73, WDR81, WFS1, WWOX, XPA, XPC, ZFR, ZFYVE26, ZFYVE27, ZNF423, ZNF592

Gene set #3: Neurodegenerative disease gene set (n=976)

AAAS, AARS, AARS2, ABCB7, ABCC9, ABCD1, ABHD12, ABHD5, ACAD9, ACADVL, ACAT1, ACBD5, ACOX1, ACP33, ACTA1, ACTB, ACTC1, ACTN2, ACVR1, ADAR, ADCK3, ADCY5, ADGRG1, ADSSL1, AFG3L2, AGL, AGRN, AHI1, AIFM1, AIMP1, AKAP9, AKR1C2, ALAS2, ALDH18A1, ALDH3A2, ALG13, ALG14, ALG2, ALG6, ALPK3, ALS2, AMACR, AMPD2, ANG, ANK2, ANKRD1, ANO10, ANO3, ANO5, ANXA11, AP1S2, AP4B1, AP4E1, AP4M1, AP4S1, AP5Z1, APOA1BP, APOE, APOPT1, APP, APTX, AR, ARG1, ARHGEF10, ARL13B, ARL3, ARL6IP1, ARMC9, ARSA, ARSI, ARX, ASAH1, ASCC1, ASPA, ATAD1, ATCAY, ATG5, ATL1, ATL3, ATM, ATN1, ATP13A2, ATP1A1, ATP1A2, ATP1A3, ATP2A1, ATP2B3, ATP2B4, ATP7A, ATP7B, ATP8A2, ATP9A, ATPAF2, ATXN1, ATXN10, ATXN2, ATXN3, ATXN7, ATXN8, ATXN8OS, AUH, B3GALNT2, B4GALNT1, B4GAT1, B9D1, BAG3, BCAP31, BCS1L, BEAN1, BICD2, BIN1, BOLA3, BSCL2, BTD, BVES, C10orf2, C11orf73, C12orf65, C19orf12, C5orf42, C9orf72, CA2, CA8, CACNA1A, CACNA1B, CACNA1C, CACNA1G, CACNA1S, CACNA2D2, CACNB2, CACNB4, CALM1, CALM2, CALR3, CAMTA1, CAPN1, CAPN3, CASK, CASQ1, CASQ2, CAV3, CC2D2A, CCDC78, CCDC88C, CCT5, CDK16, CDKL5, CEP104, CEP120, CEP290, CEP41, CFL2, CHAT, CHCHD10, CHCHD2, CHKB, CHMP1A, CHMP2B, CHRNA1, CHRNB1, CHRND, CHRNE, CHRNG, CIC, CIZ1, CLCN1, CLCN2, CLN3, CLN5, CLN6, CLN8, CLP1, CLTCL1, CNBP, CNTN1, CNTNAP1, COA7, COASY, COG5, COL12A1, COL13A1, COL25A1, COL4A1, COL4A2, COL6A1, COL6A2, COL6A3, COLQ, COQ2, COQ6, COQ8A, COQ9, COX10, COX15, COX20, COX6A1, CP, CPT1C, CPT2, CRYAB, CSF1R, CSNK1D, CSPP1, CSRP3, CSTB, CTC1, CTDP1, CTNNA3, CTSA, CTSD, CTSF, CWF19L1, CYP27A1, CYP2U1, CYP7B1, D2HGDH, DAB1, DAG1, DARS, DARS2, DCAF17, DCAF8, DCTN1, DCX, DDB2, DDC, DDHD1, DDHD2, DES, DGAT2, DGUOK, DHTKD1, DLAT, DMD, DMPK, DMXL2, DNAJB2, DNAJB6, DNAJC12, DNAJC13, DNAJC19, DNAJC3, DNAJC5, DNAJC6, DNM2, DNMT1, DOK7, DOLK, DPAGT1, DPM1, DPM2, DPM3, DPYD, DSC2, DSG2, DSP, DST, DSTYK, DTNA, DUX4, DYNC1H1, DYSF, EARS2, EEF2, EGR2, EIF2AK2, EIF2B1, EIF2B2, EIF2B3, EIF2B4, EIF2B5, ELOVL4, ELOVL5, EMD, ENO3, ENTPD1, EPM2A, EPT1, ERBB3, ERBB4, ERCC2, ERCC3, ERCC4, ERCC5, ERCC6, ERCC8, ERLIN1, ERLIN2, ETFA, ETFB, ETFDH, EXOSC3, EXOSC8, EXT1, EYA4, FA2H, FAM111B, FAM126A, FAM134B, FARS2, FASTKD2, FBLN5, FBXO38, FBXO7, FGD4, FGF14, FHL1, FIG4, FKRP, FKTN, FLAD1, FLNA, FLNC, FLRT1, FLVCR1, FLVCR2, FMR1, FOLR1, FOXC1, FOXG1, FRRS1L, FTL, FUCA1, FUS, FXN, GAA, GAD1, GAK, GALC, GAN, GARS, GATAD1, GBA, GBA2, GBE1, GCDH, GCH1, GDAP1, GFAP, GFM1, GFPT1, GJA1, GJA5, GJB1, GJB3, GJC2, GLA, GLB1, GLDC, GLE1, GLRA1, GLRB, GLRX5, GLTP, GMPPB, GNAL, GNAO1, GNB1, GNB4, GNE, GOLGA2, GOSR2, GPAA1, GPD1L, GPR56, GPT2, GRID2, GRM1, GRN, GYG1, GYS1, HACE1, HARS, HCN4, HEPACAM, HEXA, HEXB, HINT1, HK1, HMBS, HNRNPA1, HNRNPA2B1, HNRNPDL, HOXD10, HPCA, HPRT1, HRAS, HSD17B4, HSPB1, HSPB3, HSPB8, HSPD1, HSPG2, HTRA1, HTT, HYLS1, IBA57, IDS, IFIH1, IFRD1, IGHMBP2, IKBKAP, ILK, INF2, INPP5E, INPP5K, IRF2BPL, ISCA2, ISCU, ISPD, ITGA7, ITM2B, ITPR1, JAM3, JPH2, JPH3, JUP, KARS, KBTBD13, KCNA1, KCNA2, KCNA5, KCNC1, KCNC3, KCND3, KCNE1, KCNE2, KCNE3, KCNH2, KCNJ10, KCNJ18, KCNJ2, KCNJ5, KCNMA1, KCNMA1, KCNQ1, KCNQ2, KCNQ3, KCNT1, KCTD17, KCTD7, KDM5C, KIAA0196, KIAA0226, KIAA0556, KIAA0586, KIAA0753, KIDINS220, KIF1A, KIF1B, KIF1C, KIF21A, KIF5A, KIF7, KLC2, KLC4, KLHL40, KLHL41, KLHL9,

KMT2B, KY, L1CAM, L2HGDH, LAMA2, LAMA4, LAMA5, LAMB1, LAMB2, LAMP2, LARGE, LDB3, LDHA, LIMS2, LITAF, LMNA, LMNB1, LMOD3, LPIN1, LRP4, LRRK2, LRSAM1, LYRM7, LYST, MAG, MAN2B1, MAP3K20, MAPT, MARS, MARS2, MATR3, MCOLN1, MECP2, MECR, MED25, MEF2C, MEGF10, MFF, MFN2, MFSD8, MIB1, MKS1, MLC1, MMACHC, MME, MORC2, MPLKIP, MPV17, MPZ, MRE11, MRE11A, MRPL3, MRPL44, MRPS16, MSTN, MSTO1, MT-ATP6, MT-ATP8, mt-ND6, MTFMT, MTM1, MTMR2, MTO1, MTPAP, MTTP, MURC, MUSK, MUT, MVK, MYBPC1, MYBPC3, MYH2, MYH3, MYH6, MYH7, MYH8, MYL2, MYL3, MYL4, MYLK2, MYMK, MYO18B, MYO9A, MYOT, MYOZ2, MYPN, NAGLU, NARS2, NAXE, NDE1, NDRG1, NDUFA2, NDUFAF1, NDUFAF3, NDUFS1, NDUFS2, NDUFS4, NDUFS7, NDUFS8, NDUFV1, NEB, NEFH, NEFL, NEK1, NEU1, NEXN, NFU1, NGF, NHLRC1, NIPA1, NKX2-1, NKX6-2, NOP56, NOTCH3, NPC1, NPC2, NPHP1, NPPA, NT5C2, NTRK1, NTRK2, NUBPL, NUP155, NUP62, OBFC1, OCLN, OCRL, OFD1, OPA1, OPA3, OPHN1, OPTN, ORAI1, PABPN1, PAFAH1B1, PANK2, PARK2, PARK7, PAX2, PAX6, PC, PCCA, PCCB, PCDH12, PCLO, PCNA, PCYT2, PDE10A, PDE6D, PDE8B, PDGFB, PDGFRB, PDHA1, PDHX, PDK3, PDSS1, PDSS2, PDYN, PEX1, PEX10, PEX11B, PEX12, PEX13, PEX14, PEX16, PEX19, PEX2, PEX26, PEX3, PEX5, PEX6, PEX7, PFKM, PFN1, PGAM2, PGAP1, PGK1, PGM1, PHGDH, PHKA1, PHOX2A, PHYH, PIBF1, PIEZO2, PIK3R5, PINK1, PIP5K1C, PKP2, PLA2G6, PLAA, PLEC, PLEKHG2, PLEKHG4, PLEKHG5, PLN, PLP1, PMM2, PMP2, PMP22, PMPCA, PNKD, PNKP, PNPLA2, PNPLA6, PNPLA8, POGLUT1, POLG, POLG2, POLR1A, POLR1C, POLR3A, POLR3B, POMGNT1, POMGNT2, POMK, POMT1, POMT2, PPP2R2B, PPT1, PRDM12, PRDM16, PRDX3, PREPL, PRF1, PRICKLE1, PRKAG2, PRKCG, PRKN, PRKRA, PRNP, PRPH, PRPS1, PRRT2, PRUNE1, PRX, PSAP, PSAT1, PSEN1, PSEN2, PTPLA, PTRF, PTRH2, PTS, PUS1, PYCR2, PYGM, PYROXD1, QDPR, RAB11B, RAB12, RAB39B, RAB3A, RAB3GAP2, RAB7A, RAF1, RAPSN, RARS, RARS2, RBCK1, RBM20, RBM7, REEP1, REEP2, RELN, RETREG1, RNASEH1, RNASEH2A, RNASEH2B, RNASEH2C, RNASET2, RNF168, RNF170, RNF216, RNU12, RPGRIP1L, RPH3A, RRM2B, RTN2, RUBCN, RYR1, RYR2, RYR3, SACS, SAMD9L, SAMHD1, SAR1B, SBF1, SBF2, SCN11A, SCN1A, SCN1B, SCN2B, SCN3B, SCN4A, SCN4B, SCN5A, SCN8A, SCN9A, SCO1, SCO2, SCP2, SCYL1, SDHA, SDHAF1, SDHB, SDHD, SELENON, SEPSECS, SEPT9, SERAC1, SETX, SGCA, SGCB, SGCD, SGCE, SGCG, SGPL1, SH3TC2, SIGMAR1, SIL1, SLC12A6, SLC13A5, SLC16A1, SLC16A2, SLC17A5, SLC18A3, SLC19A3, SLC1A3, SLC1A4, SLC20A2, SLC22A5, SLC25A1, SLC25A12, SLC25A15, SLC25A20, SLC25A4, SLC25A42, SLC25A46, SLC2A1, SLC30A10, SLC33A1, SLC39A14, SLC52A2, SLC52A3, SLC5A7, SLC6A3, SLC6A4, SLC6A5, SLC9A1, SLC9A6, SMCHD1, SMN1, SMPD1, SNAP25, SNCA, SNORD118, SNTA1, SNX14, SOD1, SON, SOX10, SPART, SPAST, SPEG, SPG11, SPG20, SPG21, SPG7, SPR, SPTAN1, SPTBN2, SPTBN4, SPTLC1, SPTLC2, SQSTM1, SRD5A3, STAC3, STIM1, STUB1, SUCLA2, SUMF1, SURF1, SYNE1, SYNE2, SYNJ1, SYT14, SYT2, TACO1, TAF1, TARDBP, TAZ, TBC1D24, TBCE, TBK1, TBP, TCAP, TCTN1, TCTN2, TCTN3, TDP1, TDP2, TECPR2, TECRL, TFG, TGFB3, TGM6, TH, THAP1, TIA1, TIMM8A, TK2, TMEM106B, TMEM138, TMEM216, TMEM231, TMEM237, TMEM240, TMEM43, TMEM5, TMEM65, TMEM67, TMPO, TNNC1, TNNI2, TNNI3, TNNT1, TNNT2, TNNT3, TNPO3, TOR1A, TOR1AIP1, TPM1, TPM2, TPM3, TPP1, TRAPPC11, TRDN, TREM2, TREX1, TRIM2, TRIM32, TRIM54, TRIM63, TRIP4, TRPC3, TRPV4, TSEN2, TSEN34, TSEN54, TSFM, TTBK2, TTC19, TTN, TTPA, TTR, TUBA1A, TUBA4A, TUBA8, TUBB2B, TUBB3, TUBB4A, TUFM, TWNK, TYMP, TYROBP, UBA1, UBAP1, UBQLN2, UBR4, UBTF, UCHL1, UNC13B, UNC13D, USP8, VAC14, VAMP1, VAMP2, VAPB, VARS2, VCL, VCP, VLDLR, VMA21, VPS11, VPS13A, VPS13C, VPS13D, VPS35, VPS37A, VPS53, VRK1, VWA3B, WARS, WASHC5, WDR45, WDR45B, WDR48, WDR73, WDR81, WFS1, WNK1, WWOX, XK, XPA, XPC, XPR1, XRCC1, YARS, YARS2, ZEB2, ZFHX2, ZFR, ZFYVE26, ZFYVE27, ZNF423, ZNF592

SUPPLEMENT 3: Workflow of next-generation sequencing analysis and data interpretation

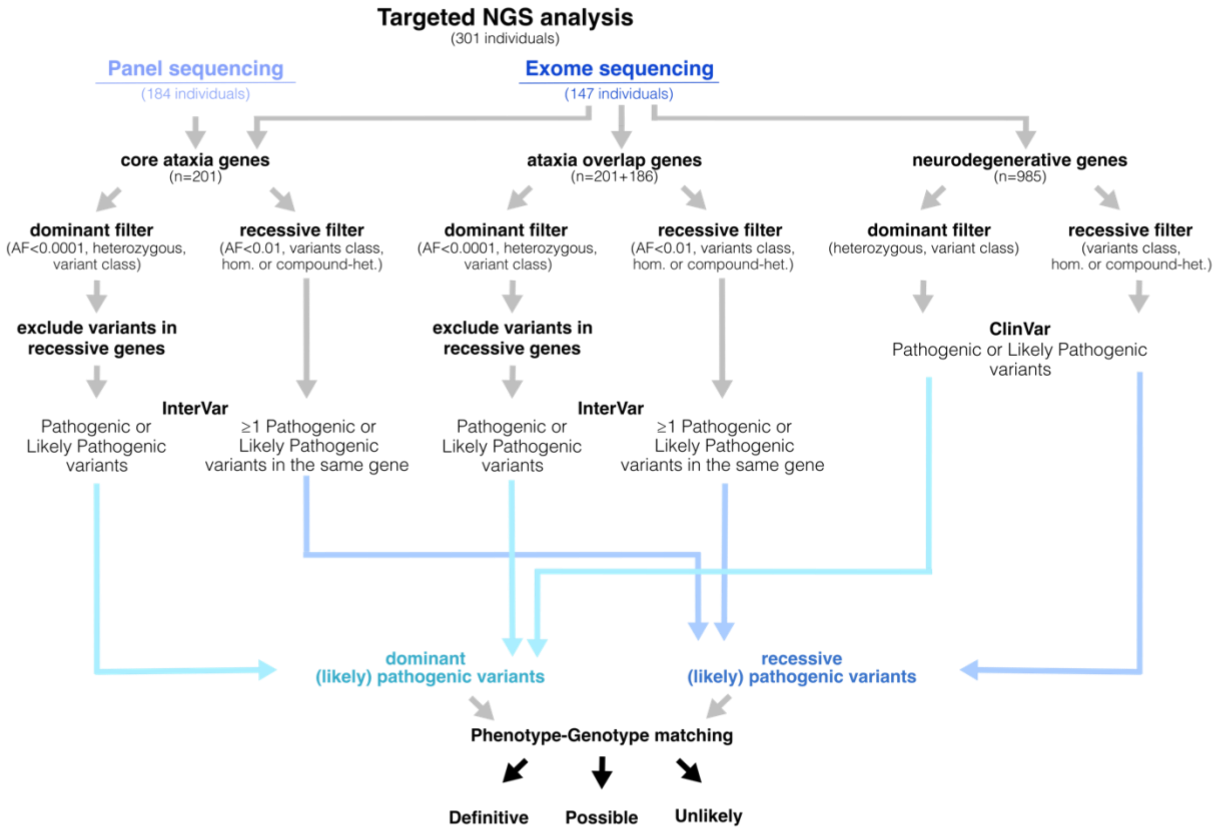

Supplement 3: Graphical depiction of the targeted NGS data analysis workflow employed in this study, making use of exome sequencing and large panel sequencing data with analysis focused on specific gene sets (i) core ataxia genes (n=201), (ii) ataxia-overlap genes (n=201+186) and (iii) neurodegenerative genes (n=985) (see also Supplement 1), with separate filters for dominant and recessive inheritance.

SUPPLEMENT 4: cohort characteristics

	TOTAL	SAOA	MSA-C_{CP}	SIGNIFICANCE
**INDIVIDUALS**	377	229	148	-
**MALE**	213	131	82	ns
**FEMALE**	164	98	66	
**AGE AT ONSET (Y)**	57 (IQR 51-63)	57 (IQR 50-65)	57 (IQR 52-63)	P = .463
**AGE AT LAST VISIT (Y)**	65 (IQR 59-72)	66 (IQR 60-74)	63 (IQR 58-68)	P < .001
**DISEASE DURATION (Y)**	6.7 (IQR 4.8-10.7)	7.7 (IQR 4.9-13.1)	6.1 (IQR 4.6-7.5)	P < .001
**DISEASE SEVERITY (SARA POINTS)**	15 (IQR 11-20)	13 (IQR 9.5-17)	19 (IQR 14-24.5)	P < .001

Table Supplement 4: Cohort demographics, Abbreviations: F – female; IQR – interquartile range; M - male; MSA-C_{cp} – patients meeting the criteria of clinically probable multiple system atrophy cerebellar type; ns – not significant; SAOA – sporadic adult-onset ataxia of unknown etiology.

SUPPLEMENT 5: Individuals with pathogenic *FGF14*-GAA repeat expansion (≥250 repeats)

Individual #	Sex	Age at onset	sporadic ataxia cluster	*FGF14* repeat count – long allele	*FGF14* repeat count – short allele	*FGF14* repeat motif	Zygosity	Age at assessment	SARA
P41	M	65	SAOA	271	58	GAA	heterozygous	65	2.5
P42	F	57	SAOA	273	11	GAA	heterozygous	81	13
P1	F	62	MSA-C_{cp}	311	87	GAA	heterozygous	65	9
P43	M	69	SAOA	303	66	GAA	heterozygous	75	13
P44	M	60	SAOA	333	62	GAA	heterozygous	84	8
P45	F	64	SAOA	282	7	GAA	heterozygous	72	12
P46	M	66	SAOA	400	43	GAA	heterozygous	83	11.5
P47	M	58	SAOA	349	8	GAA	heterozygous	73	23
P48	M	64	SAOA	450	8	GAA	heterozygous	73	11
P49	M	56	SAOA	387	9	GAA	heterozygous	62	7.5
P50	M	57	SAOA	402	8	GAA	heterozygous	62	9
P51	M	60	SAOA	374	8	GAA	heterozygous	77	17.5
P52	M	56	SAOA	427	9	GAA	heterozygous	62	5
P2	F	56	MSA-C_{cp}	314	10	GAA	heterozygous	61	25
P53	F	58	SAOA	296	8	GAA	heterozygous	65	11.5
P3	M	61	MSA-C_{cp}	289	9	GAA	heterozygous	63	19
P54	F	73	SAOA	319	15	GAA	heterozygous	75	14
P55	M	70	SAOA	331	10	GAA	heterozygous	73	18
P56	M	58	SAOA	257	155	GAA	heterozygous	79	34

Individual #	Sex	Age at onset	sporadic ataxia cluster	*FGF14* repeat count – long allele	*FGF14* repeat count – short allele	*FGF14* repeat motif	Zygosity	Age at assessment	SARA
P57	F	69	SAOA	351	142	GAA	heterozygous	80	19
P58	M	68	SAOA	384	10	GAA	heterozygous	74	14
P59	M	48	SAOA	325	9	GAA	heterozygous	63	13
P60	F	58	SAOA	483	8	GAA	heterozygous	73	5
P61	F	74	SAOA	262	208	GAA	heterozygous	81	16
P4	M	67	MSA-C_{cp}	329	9	GAA	heterozygous	72	8
P5	F	63	MSA-C_{cp}	309	9	GAA	heterozygous	70	17.5
P62	M	74	SAOA	329	9	GAA	heterozygous	78	9
P63	M	60	SAOA	373	45	GAA	heterozygous	69	17
P64	M	50	SAOA	303	32	GAA	heterozygous	65	11
P6	M	55	MSA-C	274	174	GAA	heterozygous	59	19
P65	F	73	SAOA	276	252	GAA	**homozygous**	79	27
P66	M	74	SAOA	260	154	GAA	heterozygous	79	18.5
P67	M	45	SAOA	288	9	GAA	heterozygous	68	10
P7	M	60	MSA-C_{cp}	273	191	GAA	heterozygous	68	15.5
P68	F	61	SAOA	345 - GAA	319 - non-GAA	GAA	heterozygous	67	11.5
P32	M	68	SAOA	294	16	GAA	heterozygous	73	9
P8	M	77	MSA-C_{cp}	409	8	GAA	heterozygous	77	11
P69	F	66	SAOA	286	16	GAA	heterozygous	76	15
P70	F	56	SAOA	393	8	GAA	heterozygous	75	7.5
P71	F	68	SAOA	346	8	GAA	heterozygous	83	13

Individual #	Sex	Age at onset	sporadic ataxia cluster	*FGF14* repeat count – long allele	*FGF14* repeat count – short allele	*FGF14* repeat motif	Zygosity	Age at assessment	SARA
P72	M	74	SAOA	291	77	GAA	heterozygous	79	7
P40	M	59	SAOA	274	35	GAA	heterozygous (also POLG mutations)	73	22
P73	F	57	SAOA	418	8	GAA	heterozygous	70	20
P74	F	55	SAOA	360	21	GAA	heterozygous	62	6
P9	M	50	MSA-C_{cp}	402	53	GAA	heterozygous	76	7.5

Abbreviations: MSA-C_{cp}- clinically probable multiple system atrophy cerebellar type; SAOA – sporadic adult-onset ataxia of unknown etiology; SARA - Scale for the assessment and rating of ataxia

SUPPLEMENT 6: Individuals with intermediate *FGF14*-GAA repeat expansion (200-249 repeats)

Individual #	Sex	Age at onset	sporadic ataxia cluster	*FGF14* repeat count – long allele	*FGF14* repeat count – short allele	*FGF14* repeat motif	Zygosity	Age at assessment	SARA
P30	F	52	SAOA	213	9	GAA	heterozygous	58	26
P75	M	52	SAOA	205	32	GAA	heterozygous	58	10
P76	M	62	SAOA	218	9	GAA	heterozygous	71	13
P77	F	49	SAOA	236 - GAA	339 – non-GAA	GAA/ non-GAA	heterozygous (also *RFC1*)	60	10
P78	F	49	MSA-C_{cp}	206	16	GAA	heterozygous	53	21.5
P18	M	57	MSA-C_{cp}	244	138	GAA	heterozygous	60	18.5
P79	F	42	SAOA	224	8	GAA	heterozygous	67	14.5
P80	F	68	SAOA	208	9	GAA	heterozygous	77	15
P81	F	60	SAOA	241	17	GAA	heterozygous	77	18
P82	M	53	MSA-C_{cp}	242	10	GAA	heterozygous	61	27
P83	M	54	SAOA	206	15	GAA	heterozygous	79	12
P84	M	53	MSA-C_{cp}	223	10	GAA	heterozygous	55	19
P85	M	55	SAOA	228	35	GAA	heterozygous	80	16
P86	F	66	SAOA	207	8	GAA	heterozygous	70	17
P87	F	54	MSA-C_{cp}	216	168	GAA	heterozygous	59	26
P88	F	65	SAOA	206	132	GAA	heterozygous	78	19

Abbreviations: MSA-C_{cp} – clinically probable multiple system atrophy cerebellar type; SAOA - sporadic adult-onset ataxia of unknown etiology; SARA - Scale for the assessment and rating of ataxia

SUPPLEMENT 7: Individuals with biallelic *RFC1* repeat expansions

Individual #	Sex	Age at onset	Sporadic ataxia cluster	*RFC1* repeat motif	Zygosity	Age at last assessment	SARA
P89	M	56	SAOA	AAGGG	biallelic	72	17
P90	F	55	SAOA	AAGGG	biallelic	60	2
P91	F	61	SAOA	AAGGG	biallelic	66	13.5
P92	F	50	SAOA	AAGGG	biallelic	73	31
P93	F	44	SAOA	AAGGG	biallelic	66	21
P10	F	46	MSA-C_{cp}	AAGGG	biallelic	61	14
P11	M	47	MSA-C_{cp}	AAGGG	biallelic	61	9.5
P94	M	55	SAOA	AAGGG	biallelic	68	28
P12	F	49	MSA-C_{cp}	AAGGG	biallelic	64	22.5
P95	M	50	SAOA	AAGGG	biallelic	58	22
P96	M	66	SAOA	AAGGG	biallelic	77	18.5
P77	F	49	SAOA	AAGGG	biallelic (also *FGF14*-GAA 236 repeats)	60	10
P97	F	54	SAOA	AAGGG	biallelic	72	18.5
P98	M	62	SAOA	AAGGG	biallelic	79	13
P31	M	45	SAOA	AAGGG	biallelic	60	14.5
P99	F	47	SAOA	AAGGG	biallelic	58	11.5
P100	F	67	SAOA	AAGGG	biallelic	81	22

Abbreviations: MSA-C_{cp} – clinically probable multiple system atrophy cerebellar type; SAOA - sporadic adult-onset ataxia of unknown etiology; SARA - Scale for the assessment and rating of ataxia

SUPPLEMENT 8: Clinico-genetic case vignettes of specific patients: patients with *ATM* and *SOD1* variants

A. Patients with *ATM* variants

Patient 16: two *ATM* variants (NM_000051.4: c.94C>T (p.Arg32Cys) and c.1339C>T (p.Arg447Ter)), and meeting the MSA-C_{cp} diagnostic criteria

At the last follow-up, a 54-year-old female patient, who first experienced symptoms at age 47, presented with primarily cerebellar involvement, including postural and gait ataxia, fine motor coordination problems, and dysarthria. The patient's SARA score at age 53 was 17.5. Although she initially showed no signs of afferent or pyramidal involvement, later stages included broadened reflex zones and urinary urge incontinence. Spasticity or Babinski sign were not present. Hypokinetic symptoms evolved over the disease course, but she never developed chorea. Progression included the need for a walking frame already 5 years after disease onset (i.e. by age 52 years), and already 7 years after disease onset (age 54 years) she required intermittent lateral support to stand. Her presented with highly unsteady ataxic gait and could not perform tandem stance, heel stance, or straight-line walking. Her cMRI showed the typical combination of MRI findings for MSA-C, including – in addition to cerebellar atrophy- the hot cross bun sign, atrophy of the pons, and atrophy the middle cerebellar peduncles, all of them progressing over time (see Supplement 8 - Figure 1 below).

The patient has no family history of neurological disease. There were no non-neurological features as often observed at least in classic AT phenotypes (e.g. teleangiectasias, immunodeficiency, leukemia, etc), that could substantiate the specific *ATM* genetic finding, but these findings would also often be missing in variant (i.e. late-onset) AT phenotypes. Alpha-Fetoprotein (AFP) in blood were not assessed.

The c.1339C>T, p.Arg447Ter *ATM* variant causes premature truncation of the ATM protein, likely to be degraded via nonsense-mediated decay. Loss-of-function is a well-established mechanism for *ATM* variants. The c.1339C>T variant has been described before as a known pathogenic mutation, mostly associated with classical early-onset AT, as expected based on the likely complete loss of expression from this allele. The c.94C>T, p.Arg32Cys is a missense variant with a CADD score of 26.5, and predicted to be damaging by SIFT and possibly damaging by PolyPhen2. While it is listed so far as a variant of unknown significance, the low frequency of the variant in reference database gnomAD (allele count 86- allele frequency 5.33e-5 – 0 homozygous occurrences) together with the *in-silico* predictions by several tools (CADD, SIFT, PolyPhen2) are supportive of pathogenicity. The combination of these two types of *ATM* variants- one truncating and one missense variant – would well be in line with predisposing to variant AT (rather than classic AT) disease course.

In sum, in particular the MRI signs highly characteristic of MSA (while not observed in AT) make it likely that this patient has two co-occurring, but independent findings: MSA-C and, independent thereof, two ATM variants predisposing to a variant AT disease course.

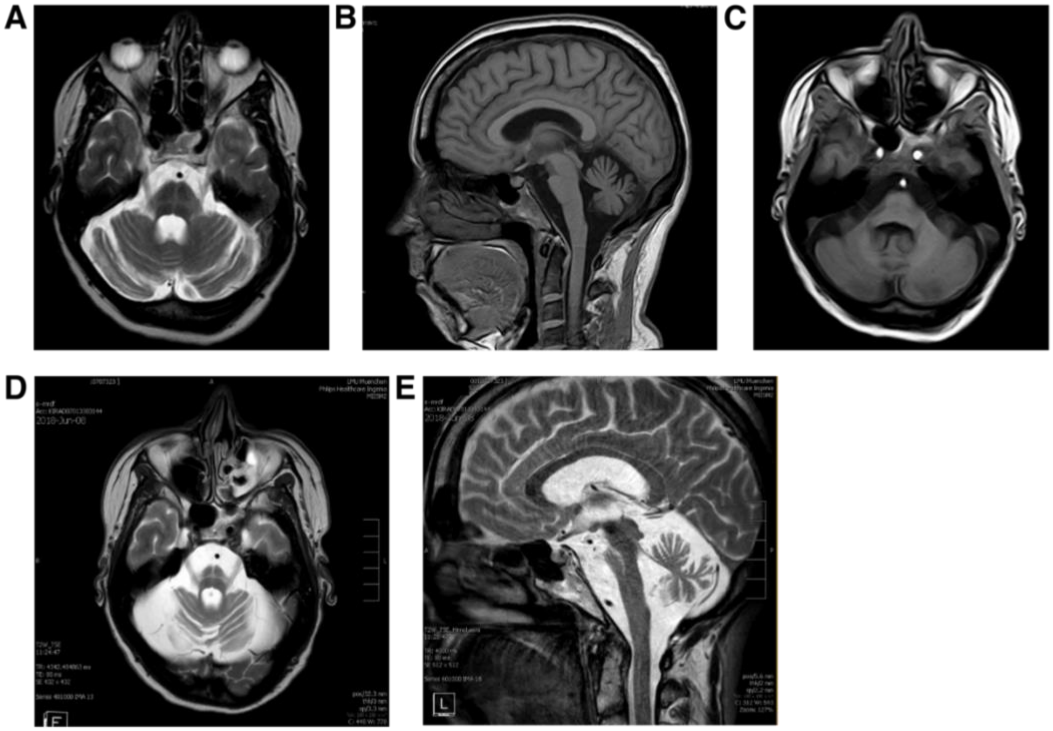

Supplement 8 - Figure 1: MRI images of patient 16 at age 49 (A-C) and age 57 (D-E). Axial T2-weighted images (A and D) show a hot cross bun sign, progressive over time. Sagittal T-weighted (B) or T2-weighted (E) images demonstrate progressive atrophy of both the cerebellum, affecting both anterior and posterior vermis, and the pons. In addition, the axial T1-weighted image (C) shows the atrophy of the middle cerebellar peduncles.

Patient 34: two *ATM* variants (NM_000051.4: c.4882A>G (p.Met1628Val) and c.8264A>C (p.Tyr2755Ser), and a late adult-onset sporadic ataxia phenotype (SAOA)

At the last follow-up, a 63-year-old female patient, who first experienced symptoms at age 47, presented with the primarily cerebellar involvement: including postural and gait ataxia accompanied by fine motor coordination problems and mild speech impairments. Oculomotor examination showed broken-up smooth pursuit, gaze-evoked nystagmus, and hypermetric saccades. The patient's SARA score after 16 years disease duration (i.e.) was 17/40 points. As extra-cerebellar signs, she showed areflexia in both the upper and lower limbs, moderate impairment in vibration sense in the lower limbs, and moderate distal muscle atrophy in the lower limbs. The patient has no family history of neurological disease. There were no non-neurological features as often observed at least in classic AT phenotypes (e.g. teleangiectasias, immunodeficiency, leukemia, etc), that could substantiate the specific *ATM* genetic finding, but these findings would also commonly be missing in variant (i.e. late-onset) AT phenotypes. The patient showed increased Alpha-Fetoprotein (AFP) level (13 µg/L; reference range <10 µg/L). While normal AFP levels do not rule out pathogenicity of *ATM* variants (in particular in the setting of variant AT patients), increased AFP levels add biomarker support for their pathogenicity.

The c.8264A>C, (p.Tyr2755Ser) *ATM* variant is a missense affecting the critical catalytic site of the protein, which has been demonstrated to have severe effects on ATM function. The variant has been described as a known pathogenic variant mutation, associated with classic early-onset AT. The c.4882A>G, (p.Met1628Val) *ATM* variant is a likely pathogenic predicted missense variant that has not been reported outside of clinical-genetic repository ClinVar, where it was reported once. *In-silico* predictions for this variant suggest a high likelihood that this variant, in

addition to its missense effect, might also result in some aberrant splicing (SpliceAI >0.93 Donor Gain), but RNA was unavailable to verify this additional prediction. The absence of the variant in gnomAD (allele count and allele frequency of 0) also supports pathogenicity of this variant.
The combination of these two types of *ATM* variants- one truncating and one missense variant (potentially leading to a leaky splice effect) – would well be in line with predisposing to variant AT (rather than classic AT) disease course.

B. Patients with *SOD1* variants

Patient 31: one *SOD1* variant (NM_000454.5: c.217G>A (p.Gly73Ser)), and a homozygous *RFC1* repeat expansion (AAGGG motif)
At the last follow-up, a 60-year-old male patient, who first experienced symptoms at age 45, presented with primarily cerebellar involvement, including postural and gait ataxia accompanied by dysarthria and Achilles tendon areflexia and patellar tendon areflexia. Oculomotor findings included broken-up smooth pursuit, slowed saccades, and ophthalmoparesis on vertical gaze. Sensory examination revealed a moderate loss of vibration sense in the distal lower limbs. Additionally, the patient experienced dysphagia. The patient's SARA score after 5 years disease duration (i.e. at age 60) was 14.5/40 points.
As motor neuron signs, he showed both upper motor neuron signs (spastic gait) and lower motor neuron signs (muscle atrophy in both the upper and lower limbs). Motor nerve conduction studies of the right peroneal nerve showed prolonged distal motor latency, reduced compound muscle action potential (CMAP), and decreased motor nerve conduction velocity (MNCV) below the fibular head. Similar findings were noted for the left tibial nerve. Sensory nerve conduction studies of the right median nerve showed reduced sensory nerve action potential (SNAP) and decreased sensory nerve conduction velocity (SNCV) with palm stimulation. No SNAP could be recorded from the right sural and ulnar nerves. The patient has no family history of neurological disease.
The c.217G>A, p.Gly73Ser *SOD1* variant is a well-known pathogenic *SOD1* missense variant, described consistently and commonly as causative for amyotrophic lateral sclerosis (motor neuron disease) (6-8). Functional studies of this mutations have demonstrated a clear effect of this missense on SOD1 function (9).The patient also carries a biallelic expansion in *RFC1* of the known pathogenic AAGGG motif, that passed the pathogenic repeat size, although exact repeat size was not determined.
Thus, in sum, the patient shows signs of both, a combined cerebellar and afferent ataxia (as characteristic for *RFC1*-associated ataxia) and for a motor neuron disease with both upper motor neuron signs (spastic gait) and lower motor neuron signs (muscle atrophy in distal limbs) (as potential, still largely oligosymptomatic signs for a *SOD1* disease).

Patient 32: one *SOD1* variant (NM_000454.5: c.160A>G (p.Asn54Asp)) and a GAA-FGF14$_{294}$ repeat expansion
At the last follow-up, a 73-year-old male patient, who first experienced symptoms at age 68, presented with primarily cerebellar involvement: including mild postural and gait ataxia accompanied with speech disturbance and only mild fine motor impairment. Oculomotor findings included broken-up smooth pursuit, slowed saccades, and hypometric saccades After 6 years disease duration (i.e. at age 73y ears), the patient had a SARA score of 9/40 points. He showed mild muscle atrophy in both the upper and lower limbs, but no other potential motor neuron signs. Sensory examination revealed a mild loss of vibration sense in the distal lower limbs.
The father of the patient died of ALS at age 68 years (but no DNA sample was available for segregation analysis of the *SOD1* variant). The c.160A>G, p.Asn54Asp *SOD1* variant is a

missense variant with a CADD score of 23.4, and predicted to be damaging by SIFT and benign by PolyPhen2. It has not been reported before, including in clinical-genetic repository ClinVar. The extreme rarity of this variant - with absence from gnomAD (allele count and frequency of 0) – combined together with the *in-silico* predictions by several tools (CADD and SIFT), and the positive family history for ALS,-are taken together supportive of pathogenicity of this *SOD1* variant. Additionally, the patient carries a heterozygous GAA-*FGF14*$_{294}$ repeat expansion which is above the currently considered pathogenic cut-off of 250 repeat units.

Thus, in sum, the patient only shows muscle atrophy in distal limbs as a potential, yet unspecific lower motor neuron sign. It has to be left open whether- in the absence of other or more specific or predominant motor neuron disease signs- these signs are indicative of a (subtle) underlying *SOD1*-associated disease component; or rather part of the GAA-*FGF14*-associated phenotype or general aging.

SUPPLEMENT 9: Diagnostic yield per SNV testing strategy

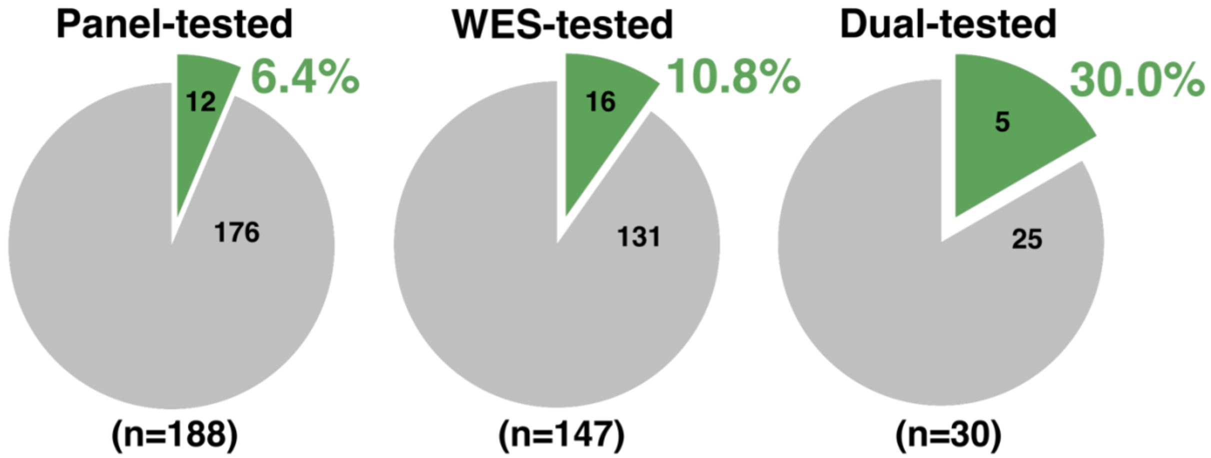

Supplement 9: Diagnostic yield of definitive/probable/unclear SNVs as identified throughout the complete SPORTAX cohort per genetic testing strategy wherein Panel-tested (n=188) contains all individuals tested by panel-only (n=158) plus dual-tested individuals (n=30). WES-tested (n=147) contains all individuals tested by WES-only (n=117) plus dual-tested individuals (n=30). Diagnostic yield reached in the dual-tested group was 5 genetic findings in 30 (=30%) Importantly, retrospective assessment of all genetic findings in dual-tested individuals showed that these could individually also have been detected through panel testing or WES testing only. Panel-tested diagnostic yield thus constitutes the 7 genetic findings obtained in panel-only indviduals plus 5 dual-tested individual (12 in 188 = 6.4%) Similarly, WES-tested diagnostic yield thus constitutes the 11 genetic findings obtained in WES-only indviduals plus 5 dual-tested individual (16 in 147 = 10.8%).

SUPPLEMENT 10: Phenotypic information patients with GAA-*FGF14* repeat expansion

Phenotypic information of patient with GAA-*FGF14* repeat expansions including sex, age at onset and age at examination, INAS count and individual INAS items

Abbreviations: BTR – Bicep tendon reflex; PTR – patellar tendon reflex; ATR – achilles tendon reflex; EPR – extensor plantar reflex; MSA-C_{cp} – clinically probable multiple system atrophy cerebellar type; NA – not available; SAOA - sporadic adult-onset ataxia of unknown etiology

SUPPLEMENT 11: Phenotypic information patients with *RFC1* repeat expansions

Phenotypic information of patient with biallelic *RFC1* repeat expansions including sex, age at onset and age at examination, INAS count and individual INAS items

Abbreviations: BTR – Bicep tendon reflex; PTR – patellar tendon reflex; ATR – achilles tendon reflex; EPR – extensor plantar reflex; HIT – head impulse testing; MSA-C_{cp} – clinically probable multiple system atrophy cerebellar type; NA – not available; SAOA - sporadic adult-onset ataxia of unknown etiology; VOR - vestibulo-ocular reflex.

SUPPLEMENTARY REFERENCES

1. Giordano I, Harmuth F, Jacobi H, Paap B, Vielhaber S, Machts J, et al. Clinical and genetic characteristics of sporadic adult-onset degenerative ataxia. Neurology. 2017;89(10):1043-9.
2. Landrum MJ, Lee JM, Benson M, Brown GR, Chao C, Chitipiralla S, et al. ClinVar: improving access to variant interpretations and supporting evidence. Nucleic acids research. 2018;46(D1):D1062-d7.
3. Harrison SM, Biesecker LG, Rehm HL. Overview of Specifications to the ACMG/AMP Variant Interpretation Guidelines. Curr Protoc Hum Genet. 2019;103(1):e93.
4. Richards S, Aziz N, Bale S, Bick D, Das S, Gastier-Foster J, et al. Standards and guidelines for the interpretation of sequence variants: a joint consensus recommendation of the American College of Medical Genetics and Genomics and the Association for Molecular Pathology. Genet Med. 2015;17(5):405-24.
5. Li Q, Wang K. InterVar: Clinical Interpretation of Genetic Variants by the 2015 ACMG-AMP Guidelines. Am J Hum Genet. 2017;100(2):267-80.
6. Muller K, Brenner D, Weydt P, Meyer T, Grehl T, Petri S, et al. Comprehensive analysis of the mutation spectrum in 301 German ALS families. J Neurol Neurosurg Psychiatry. 2018;89(8):817-27.
7. Martinelli I, Ghezzi A, Zucchi E, Gianferrari G, Ferri L, Moglia C, et al. Predictors for progression in amyotrophic lateral sclerosis associated to SOD1 mutation: insight from two population-based registries. J Neurol. 2023;270(12):6081-92.
8. Scarlino S, Domi T, Pozzi L, Romano A, Pipitone GB, Falzone YM, et al. Burden of Rare Variants in ALS and Axonal Hereditary Neuropathy Genes Influence Survival in ALS: Insights from a Next Generation Sequencing Study of an Italian ALS Cohort. Int J Mol Sci. 2020;21(9).
9. Fujisawa T, Homma K, Yamaguchi N, Kadowaki H, Tsuburaya N, Naguro I, et al. A novel monoclonal antibody reveals a conformational alteration shared by amyotrophic lateral sclerosis-linked SOD1 mutants. Ann Neurol. 2012;72(5):739-49.
