# Supplementary material for: The genetic landscape of sporadic adult-onset degenerative ataxia: a multi-modal genetic study of 377 consecutive patients from the longitudinal multi-centre SPORTAX cohort
Source: eBioMedicine. 2025 Apr 23;115:105715. doi: 10.1016/j.ebiom.2025.105715 (PMC12051541; doi:10.1016/j.ebiom.2025.105715)
Supplement: SPORTAX consortium author list [file mmc3.docx]

| **First names** | **Surnames** | **Affiliation** |
| --- | --- | --- |
| Friedrich | Erdlenbruch | Department of Neurology and Center for Translational Neuro- and Behavioral Sciences (C-TNBS), Essen University Hospital, University of Duisburg-Essen, Essen, Germany |
| Andreas | Thieme | Department of Neurology and Center for Translational Neuro- and Behavioral Sciences (C-TNBS), Essen University Hospital, University of Duisburg-Essen, Essen, Germany |
| Judith | van Gaalen | Department of Neurology, Radboud university medical center, 6525 Nijmegen, The Netherlands |
| Christos | Ganos | Department of Neurology, Charité University Hospital, Berlin, Germany |
| Jun-Suk | Kang | Department of Neurology University of Frankfurt, Frankfurt am Main, Germany |
| Marcus | Grobe-Einsler | German Center for Neurodegenerative Diseases (DZNE), Bonn, Germany, and Department of Neurology, University Hospital Bonn, Bonn, Germany |
| Ilaria | Giordano | German Center for Neurodegenerative Diseases (DZNE), Bonn, Germany, and Department of Neurology, University Hospital Bonn, Bonn, Germany |
